# Supplementary material for: The sexual and reproductive health needs and preferences of youths in sub-Saharan Africa: A meta-synthesis
Source: PLoS One. 2024 Dec 31;19(12):e0300829. doi: 10.1371/journal.pone.0300829 (PMC11687907; doi:10.1371/journal.pone.0300829)
Supplement: S1 Table — (PDF) [file pone.0300829.s003.pdf]

Data extraction summary on youth's needs and preferences regarding SRHS in sub-Saharan Africa

S/N	Tittle	Authors/ Publication date	Country of study	Name of Journal/impact factor	Context	Design	Target adolescent population	Method of data collection	Emergent themes
1.	Addressing the sexual and reproductive health needs of young adolescents living with HIV in South Africa	Vujovic, M., Struthers, H., Meyersfeld, S., Dlamini, K. and Mabizela, N (2014)	South Africa	Children and youth services review (ELSEVIER)/ 2.519	facility based	Qualitative (no mention of the specific qualitative design)	Male and female living with HIV (ALWHIV) 10-14years	Focus group discussion (FGD)	1. Service provision<br>2. Information, education, and communication<br>3. Risk perception and management
2.	The extent to which the design of available reproductive health interventions fit the reproductive health needs of adolescents living in urban poor settings of Kisenyi, Kampala, Uganda	Tuhebwe, D., Babirye, S., Ssendagire, S. and Ssengooba, F. (2021)	Uganda	BMC Public health/ 4.135 (2-year impact)	Community based	Qualitative (specific qualitative design not mentioned)	Male and female aged 15-19years	FGD	1.Sexual reproductive health (SRH) needs<br>2. Social needs.<br>3. Extent of fit between intervention design features and priority reproductive health<br>needs of the adolescents
3.	What do South African adolescents want in a sexual health service? Evidence from the South African Studies on HIV in Adolescents (SASHA) project	Smith, P., Marcus, R., Bennie, T., Nkala, B., Nchabeleng, M., Latka, M.H., Gray, G., Wallace,	South Africa	South African Medical Journal (SAMJ)/1.500	community based	Qualitative (specific qualitative design not mentioned)	Male and female aged 12-17years	FGD	1. The need for the provision of dedicated adolescent health services.<br>2. Tailored services with developmentally appropriate information.<br>3. Emphasis on the desire for confidentiality and trusting

		M. and Bekker, L.G. (2018)							relationships with health-care staff.<br>4. Availability of services and information

S/N	Tittle	Authors/ publication date	Country of study	Name of Journal/impact factor	Context	Design	Target adolescent popula	Method of data collection	Themes
4.	Engaging young people in the design of a sexual reproductive health intervention: Lessons learnt from the Yathu Yathu ("For us, by us") formative study in Zambia	Simuyaba, M., Hensen, B., Phiri, M., Mwansa, C., Mwenge, L., Kabumbu, M., Belemu, S., Shanaube, K., Schaap, A., Floyd, S. and Fidler, S. (2021)	Zambia	BMC Health Services Research (ELSEVIER)/2.908 (2-year impact)	Community based	Participatory qualitative research	Male and female, 15-24 years	FGD, observation and In-depth interviews (IDIs)	1. Community mapping: context of adolescents and young people (AYP)'s SRH<br>2. Primary discussion<br>3. Consolidation of AYP's views
5.	Youth accessing reproductive health services in Malawi: drivers, barriers, and suggestions from the perspectives of youth and parents.	, Self A., Chipokosa, S., Misomali, A., Aung, T., Harvey, S.A., Chimchere, M., Chilembwe, J., Park, L., Chalimba, C., Monjeza, E. and Kachale, F. (2018)	Malawi	Reproductive Health (BMC) /3.355 (2-year impact)	Facility based	Qualitative (specific qualitative design not mentioned)	Male and female aged 15-24years	FGD	1. Drivers of youth accessing family planning services<br>2. Barriers' youth face accessing family planning services Misconceptions and perceived side-effects<br>3. Suggestions from participants for improving family planning services
6.	Young people's perceptions of youth-oriented health services in urban Soweto, South Africa: a qualitative investigation	Schriver, B., Meagley, K., Norris, S., Geary, R. and Stein, A.D. (2014)	South Africa	Health Services Research (BMC)2.908 (2-year impact)	community based.	Ground theory as described by Borgatti and Strauss and Corbin.	Male and female youths aged 21-22years	IDIs	1. Perception of current health services.<br>2. Knowledge of youth friendly services.<br>3. Attitudes toward alternative health services.

S/N	Tittle	Authors/ publication date	Country of study	Name of Journal/impact factor	Context	Design	Target adolescent population	Method of data collection	Themes
7.	Provision of Reproductive Health Services for Adolescents -- Report of a Study in Two Local Government Areas (LGAs) of Nigeria	Olukoya, A. (1996)	Nigeria	Early Child Development and Care (Taylor & Francis)/1.206(2-year impact)	Facility based	Mixed study (Specific qualitative design not mentioned)	Out of school and in-school Male and female adolescents. Authors stated ‘’less than 15years’’	FGD and direct observation	1. Perceptions of the health problems of adolescents.<br>2. Help-seeking behaviour.
8.	Sexual and reproductive health services (SRHS) for adolescents in Enugu state, Nigeria: a mixed methods approach.	Odo, A.N., Samuel, E.S., Nwagu, E.N., Nnamani, P.O. and Atama, C.S. (2018)	Nigeria	Health Services Research (BMC)2.908 (2-year impact)	Community based	Mixed (Specific qualitative design not mentioned)	Male and female aged 12-22years	Interview (type-unclear) and FGD	1. Availability of SRHS for Adolescents.<br>2. Accessibility of SRHS to adolescents.
9.	Access to information and use of adolescent sexual reproductive health services: Qualitative exploration of barriers and facilitators in Kisumu and Kakamega, Kenya	Mutea, L., Ontiri, S., Kadiri, F., Michielesen, K. and Gichangi, P.(2020)	kenya	PLOS ONE (No impact factor documentation on the journal’s home page)	Community based.	Qualitative (specific qualitative design not mentioned)	Male and female adolescents aged 15-19 years	FGD, IDIs and Key Informant interviews (KIIs)	1. Common issues perceived to affect the health of adolescents in the community.<br>2. Barriers to access and use of SRH information and services by adolescents.<br>3. Facilitators to access and use of ASRH information

									and services.

S/ N	Tittle	Authors/ publication date	Country of study	Name of Journal/impact factor	Context	Design	Target adolescent population	Method of data collection	Themes
10	Does Making Clinic-based Reproductive Health Services More Youth-friendly Increase Service Use by Adolescents? Evidence From Lusaka, Zambia	Mmari, K.N. and Magnani, R.J. (2003)	Zambia	Journal of Adolescent Health (ELSEVIER)/7.83	Facility based	Mixed study (Specific qualitative design not mentioned)	Male and female aged 15-24years.	FGDs and interviews	1. Youth-Friendliness of Health Service.
11.	Adolescents living with HIV in the Copperbelt Province of Zambia: Their reproductive health needs and experiences.	McCarraher, D.R., Packer, C., Mercer, S., Dennis, A., Banda, H., Nyambe, N., Stalter, R.M., Mwansa, J.K., Katayamoyo, P. and Denison, J.A (2018)	Zambia	PLOS ONE	Facility based	Mixed (Specific qualitative design not mentioned)	Male and female aged 15–18years	IDIs	1. Sexual experiences including forced sex.<br>2. Sex partner age and HIV Disclosure.<br>3. Contraceptive use.<br>4. Fertility desires and prevention of mother-to-child transmission of HIV.
12.	Rights-based services for adolescents living with HIV: adolescent self-efficacy and implications for health systems in Zambia	Mburu, G., Hodgson, I., Teltschik, A., Ram, M., Haamujompa, C., Bajpai, D. and Mutali (2013)	Zambia	Reproductive Health Matters (Taylor & Francis)5.732	Facility based.	Qualitative (specific qualitative design not mentioned)	Male and female adolescents aged 10-19years	Semi-structured interviews and FGDs	1. A sense of rights, entitlement, and expectation.<br>2. Expressing unmet need.

S/N	Tittle	Authors/ publication date	Country of study	Name of Journal/impact factor	Context	Design	Target adolescent population	Method of data collection	Themes
13.	Accessing Sexual and Reproductive Health Information and Services: A Mixed Methods Study of Young Women's Needs and Experiences in Soweto, South Africa	Lince-Deroche, N., Hargey, A., Holt, K. and Shochet, T. (2015)	South Africa	African Journal of Reproductive Health. (It appears, there is no impact factor documentation on the journal website)	Facility based	Mixed (Grounded theory used for the qualitative approach)	Female adolescents 18-24 years	Semi-structured interviews.	1. Contraception: knowledge and access to services<br>2. Abortion services.<br>3. HIV testing and condom Use.<br>4. Gender-based violence.<br>5. Concerns and support systems
14.	Adolescents' Reproductive Health Problems, Service Preferences, and Accessibility.	Kimo, K. and Makuria, K. (2017)	Ethiopia	Pakistan Journal of Psychological Research (No impact factor seen)	Facility based	Mixed (Specific qualitative design not mentioned)	Male and female adolescents (12-19years)	FGD	Reproductive health service accessibility (the only qualitative theme).
15	Living as an adolescent with HIV in Zambia – lived experiences, sexual health, and reproductive needs.	Hodgson I, Julia Ross, Choolwe Haamujompa & D. Gitau-Mburu (2012)	Zambia	AIDS Care-Psychological and Socio-Medical Aspects of AIDS/HIV (Taylor & Francis) (No impact factor seen)	Facility based	Qualitative (specific qualitative design not mentioned)	Male and female adolescents aged 10-19years	semi-structured interviews and FGD	1. Informational needs.<br>2. Psychosocial and disclosure needs.<br>3. Sexual and reproductive health (SRH) needs of adolescents.<br>4. Health systems and services capacity.

S/ N	Tittle	Authors/ publication date	Country of study	Name of Journal/ impact factor	Context	Design	Target adolescent population	Method of data collection	Themes
16	Young people's perception of sexual and reproductive health services in Kenya	Godia, P.M., Olenja, J.M., Hofman, J.J. and Van Den Broek, N. (2014)	Kenya	Health Services Research (BMC)2.908 (2-year impact)	Facility based	Qualitative (Specific qualitative design not mentioned)	Male and female aged 10–24 years.	FGDs and semi-structured in-depth interviews (IDIs)	1. SRH problems faced by young people.<br>2. Addressing the SRH needs of young people.<br>3. Perceptions of existing SRH services.<br>4. Suggestions on how to improve SRH services
17.	Understanding sexual and reproductive health needs of adolescents: evidence from a formative evaluation in Wakiso district, Uganda	Atuyambe, L.M., Kibira, S.P., Bukenya, J., Muhumuza, C., Apolot, R.R. and Mulogo, E. (2015)	Uganda	Reproductive Health (BMC) /3.355 (2-year impact)	Community based	Qualitative (Specific qualitative design not mentioned)	Male and female aged 10-19years	FGD	1. Main adolescent health problems.<br>2. Adolescent SRH needs.<br>3. Health seeking behaviour and attitudes towards services.
18.	Adolescent human immunodeficiency virus self-management: Needs of adolescents in the Eastern Cape	Adams, L. and Crowley, T. (2021)	South Africa	African Journal of Primary Health Care & Family Medicine /1.04	Facility based.	qualitative exploratory-descriptive research design	Male and female adolescents aged 14 -19 years	Semi-structured interview	1. Knowledge of human immunodeficiency virus and sexual reproductive health.<br>2. Self-regulation skills.<br>3. Self-management resources.
19	Preferences for accessing sexual and reproductive health	Adhiambo, H. F, Ngayo, M. and	Kenya	PLOS ONE	Facility based	Qualitative (Specific qualitative	Male and female adolescents	IDIs and FGDs	1. Preferences of venue for receiving SRH services.

	services among adolescents and young adults living with HIV/ AIDs in Western Kenya: A qualitative study	Kwena, Z. (2022)				design not mentioned)	aged 14 -24 years		2. Preferences of qualities of SRH counsellors.
20	Pregnancy and STI/HIV prevention intervention preferences of South African adolescent girls: findings from a cultural consensus modelling qualitative study	Twitty et al. (2023)	South Africa	Culture, Health & Sexuality An International Journal for Research, Intervention and Care (Taylor & Francis)	School-based	Qualitative (Specific qualitative design not mentioned)	Females aged between 14 and 17 years	Semi-structured interviews	1. Intervention content<br>2. Intervention delivery format<br>3. Intervention setting
