# Supplementary material for: The sexual and reproductive health needs and preferences of youths in sub-Saharan Africa: A meta-synthesis
Source: PLoS One. 2024 Dec 31;19(12):e0300829. doi: 10.1371/journal.pone.0300829 (PMC11687907; doi:10.1371/journal.pone.0300829)
Supplement: S3 Table — (PDF) [file pone.0300829.s005.pdf]

S7 – List of included and excluded studies

S/N	Tittle	Authors/ Publication date	Country of study	Name of Journal/impact factor	Included/Excluded	Reason for exclusion (if excluded)
1.	Addressing the sexual and reproductive health needs of young adolescents living with HIV in South Africa	Vujovic, M., Struthers, H., Meyersfeld, S., Dlamini, K. and Mabizela, N (2014)	South Africa	Children and youth services review (ELSEVIER)/2.519	Included	Not applicable (NA)
2.	The extent to which the design of available reproductive health interventions fit the reproductive health needs of adolescents living in urban poor settings of Kisenyi, Kampala, Uganda	Tuhebwe, D., Babirye, S., Ssendagire, S. and Ssengooba, F. (2021)	Uganda	BMC Public health/ 4.135 (2-year impact)	Included	NA
3.	What do South African adolescents want in a sexual health service? Evidence from the South African Studies on HIV in Adolescents (SASHA) project	Smith, P., Marcus, R., Bennie, T., Nkala, B., Nchabeleng, M., Latka, M.H., Gray, G., Wallace, M. and Bekker, L.G. (2018)	South Africa	South African Medical Journal (SAMJ)/1.500	Included	NA

S/N	Tittle	Authors/ publication date	Country of study	Name of Journal/impact factor	Included/Excluded	Reason for exclusion (if excluded)
4.	Engaging young people in the design of a sexual reproductive health intervention: Lessons learnt from the Yathu Yathu ("For us, by us") formative study in Zambia	Simuyaba, M., Hensen, B., Phiri, M., Mwansa, C., Mwenge, L., Kabumbu, M., Belemu, S., Shanaube, K., Schaap, A., Floyd, S. and Fidler, S. (2021)	Zambia	BMC Health Services Research (ELSEVIER)/2.908 (2-year impact)	Included	NA
5.	Youth accessing reproductive health services in Malawi: drivers, barriers, and suggestions from the perspectives of youth and parents.	, Self A., Chipokosa, S., Misomali, A., Aung, T., Harvey, S.A., Chimchere, M., Chilembwe, J., Park, L., Chalimba, C., Monjeza, E. and Kachale, F. (2018)	Malawi	Reproductive Health (BMC) /3.355 (2-year impact)	Included	NA
6.	Young people's perceptions of youth-oriented health services in urban Soweto, South Africa: a qualitative investigation	Schriver, B., Meagley, K., Norris, S., Geary, R. and Stein, A.D. (2014)	South Africa	Health Services Research (BMC)2.908 (2-year impact)	Included	NA
7.	Provision of Reproductive Health Services for Adolescents -- Report of a Study in Two Local Government Areas (LGAs) of Nigeria	Olukoya, A. (1996)	Nigeria	Early Child Development and Care (Taylor & Francis)/1.206(2-year impact)	Included	NA

8.	Sexual and reproductive health services (SRHS) for adolescents in Enugu state, Nigeria: a mixed methods approach.	Odo, A.N., Samuel, E.S., Nwagu, E.N., Nnamani, P.O. and Atama, C.S. (2018)	Nigeria	Health Services Research (BMC)2.908 (2-year impact)	Included	NA
9.	Access to information and use of adolescent sexual reproductive health services: Qualitative exploration of barriers and facilitators in Kisumu and Kakamega, Kenya	Mutea, L., Ontiri, S., Kadiri, F., Michielesen, K. and Gichangi, P.(2020)	kenya	PLOS ONE (No impact factor documentation on the journal's home page)	Included	

**S/N**	**Tittle**	**Authors/ publication date**	**Country of study**	**Name of Journal/impact factor**	**Included/Excluded**	**Reason for exclusion (if excluded)**
10	Does Making Clinic-based Reproductive Health Services More Youth-friendly Increase Service Use by Adolescents? Evidence From Lusaka, Zambia	Mmari, K.N. and Magnani, R.J. (2003)	Zambia	Journal of Adolescent Health (ELSEVIER)/7.83	Included	N'A

11.	Adolescents living with HIV in the Copperbelt Province of Zambia: Their reproductive health needs and experiences.	McCarraher, D.R., Packer, C., Mercer, S., Dennis, A., Banda, H., Nyambe, N., Stalter, R.M., Mwansa, J.K., Katayamoyo, P. and Denison, J.A (2018)	Zambia	PLOS ONE	Included	N/A
12.	Rights-based services for adolescents living with HIV: adolescent self-efficacy and implications for health systems in Zambia	Mburu, G., Hodgson, I., Teltschik, A., Ram, M., Haamujompa, C., Bajpai, D. and Mutali (2013)	Zambia	Reproductive Health Matters (Taylor & Francis)5.732	Included	N/A

**S/N**	**Tittle**	**Authors/ publication date**	**Country of study**	**Name of Journal/impact factor**	**Included/Excluded**	**Reason for exclusion (if excluded)**
13.	Accessing Sexual and Reproductive Health Information and Services: A Mixed Methods Study of Young Women's Needs and Experiences in Soweto, South Africa	Lince-Deroche, N., Hargey, A., Holt, K. and Shochet, T. (2015)	South Africa	African Journal of Reproductive Health. (It appears, there is no impact factor documentation on the journal website)	Included	N/A
14.	Adolescents' Reproductive Health Problems, Service Preferences, and Accessibility.	Kimo, K. and Makuria, K. (2017)	Ethiopia	Pakistan Journal of Psychological Research (No impact factor seen)	Included	N/A
15	Living as an adolescent with HIV in Zambia – lived experiences, sexual health, and reproductive needs.	Hodgson I, Julia Ross, Choolwe Haamujompa & D. Gitau-Mburu (2012)	Zambia	AIDS Care- Psychological and Socio-Medical Aspects of AIDS/HIV (Taylor &	Included	N/A

				Francis) (No impact factor seen)		

S/N	Tittle	Authors/ publication date	Country of study	Name of Journal/ impact factor	Included/Excluded	Reason for exclusion (if excluded)
16	Young people's perception of sexual and reproductive health services in Kenya	Godia, P.M., Olenja, J.M., Hofman, J.J. and Van Den Broek, N. (2014)	Kenya	Health Services Research (BMC)2.908 (2-year impact)	Included	N/A
17.	Understanding sexual and reproductive health needs of adolescents: evidence from a formative evaluation in Wakiso district, Uganda	Atuyambe, L.M., Kibira, S.P., Bukenya, J., Muhumuza, C., Apolot, R.R. and Mulogo, E. (2015)	Uganda	Reproductive Health (BMC) /3.355 (2-year impact)	Included	N/A
18.	Adolescent human immunodeficiency virus self-management: Needs of adolescents in the Eastern Cape	Adams, L. and Crowley, T. (2021)	South Africa	African Journal of Primary Health Care & Family Medicine /1.04	Included	N/A
19	Preferences for accessing sexual and reproductive health services among adolescents and young adults living with HIV/ AIDs in Western Kenya: A qualitative study	Adhiambo, H. F, Ngayo, M. and Kwena, Z. (2022)	Kenya	PLOS ONE	Included	N/A
20	Pregnancy and STI/HIV prevention intervention preferences of South African adolescent girls: findings from a cultural consensus modelling qualitative study	Twitty et al. (2023)	South Africa	Culture, Health & Sexuality An International Journal for Research, Intervention and Care (Taylor & Francis)	Included	N/A
21	Reproductive health needs of young persons in markets and motor parks in south west Nigeria	Dare OO; Oladepo O , Cleland JG , Badru OB	Nigeria	African Journal of Medicine and Medical Sciences	Excluded	This article passed our initial screening based on its title and abstract, which aligned with our inclusion criteria.

						However, attempted to access the full text through open access platforms or our institutional subscriptions.<br>As well. the article lacked any contact information for the authors, including email addresses, which prevented us from reaching out directly to request the full text. We explored all available avenues to access the full text through our institutional resources, but were unsuccessful in obtaining it. Hence, was excluded.
22	Addressing the Sexual and Reproductive Health Needs of Young People in Ethiopia: An Analysis of the Current Situation	Nigina Muntean, Worknesh Kereta and Kirstin R Mitchell (2015)	Ethiopia	African Journal of Reproductive Health	Excluded	Full article retrieved but excluded because it targeted health care professionals primarily via key informant interviews.
23	Sexual and reproductive health priorities of adolescent girls in Lagos, Nigeria: findings from free-listing interviews	Juliet Iwelunmor /Sarah Blackstone / Ucheoma Nwaozuru / Donaldson Conserve /Patricia Iwelunmor4 / John E. Ehiri (2018)	Nigeria	International Journal of Adolescent Medicine and Health.	Excluded	Full article retrieved but data analysed quantitatively. No qualitative data.
24	What is Youth-Friendly? Adolescents' Preferences for Reproductive Health Services in Kenya and Zimbabwe	Annabel SErulkar', Charles Onoka and AlfordPhi (2022)	Zimbabwe	Women's Health and Action Research Centre (WHARC)	Excluded	Quantitative methods utilised
