# Supplementary material for: The sexual and reproductive health needs and preferences of youths in sub-Saharan Africa: A meta-synthesis
Source: PLoS One. 2024 Dec 31;19(12):e0300829. doi: 10.1371/journal.pone.0300829 (PMC11687907; doi:10.1371/journal.pone.0300829)
Supplement: S4 Table — (PDF) [file pone.0300829.s006.pdf]

Full data extraction table based on the data extraction tool used (Noyes and Lewin, 2011)

A

Study reference	Title	Year	Authors	Setting	Sample	Aims	Study Design	Phenomenon Explored	Findings
01<br><br>(eligible)	Addressing the sexual and reproductive health needs of young adolescents living with HIV in South Africa	2014	Vujovic, M., Struthers, H., Meyersfeld, S., Dlamini, K. and Mabizela, N.	2 clinics in Gauteng town and Limpopo province in South Africa	HIV positive adolescents 10-14years and providers<br>Total samples= 27 adolescents (17girls; 10 boys) and 9 service providers (3 doctors, a nurse, 2 social workers and 3 counsellors)<br>Samples were from primary or secondary schools	Explored programmatic approaches to the sexual and reproductive health of very young adolescents (VYAs)	Qualitative. FGD (2 sessions) for adolescents (after school FGD)<br>- Interviews (one-to-one, face-to- face or telephone) for providers	Sexual and reproductive health needs of young adolescents living with HIV	Lack of SRH programmes for HIV positive adolescents such as education on SRH issues, developing bodies, family planning<br>-Adolescents needed separate adolescent clinics because adults are rude and impatient (lack of access to youth friendly services); were reluctant to discuss SRH issues with parents and care givers because of fear as it is considered a taboo.<br>-Adolescents had need for age - appropriate information including sexual decision making and information related to HIV. Relied on peers, media, and life orientation classes at school for this information.<br>-On accessing gender-specific information- adolescents preferred discussing with same-sex groups and same-sex facilitators<br>-Boys preferred information on how to use condoms- *"make their own condoms – using bread bags – so they won't be seen trying to get condoms in public" (Male FGD participant, Groblersdal).*<br>while girls preferred information on menstruation, relationship, safe sex and hygiene.<br>*"if a boy is forcing you to let him touch your private parts what must you do?" (Female FGD)*<br>-Both boys and girls wanted to know about the developmental changes in the opposite sex.<br>Need for support- *"You need support from people who will understand your condition" (Male FGD participant)*<br>Poor parent child communication:<br>*"…my mum wrote information in a book telling me how I came into this world, but we never had a face to face conversation about that…she is too shy to speak to me about my development" (Male FGD participant, Alexandra Township).*

A

Study reference	Title	Year	Authors	Setting	Sample	Aims	Study Design	Phenomenon Explored	Findings
									-Need for life skills programmes identified due to vulnerability of HIV infected adolescents to alcohol and drug abuse, rape.<br>-Stigma and discrimination impacted negatively on participant's (adolescents) mental health.<br>-Providers were not knowledgeable and had limited skills in addressing needs of the adolescents.
02<br><br>(eligible)	The extent to which the design of available reproductive health interventions fit the reproductive health needs of adolescents living in urban poor settings of Kisenyi, Kampala, Uganda	2021	Tuhebwe, D., Babirye, S., Ssendagire, S. and Ssengooba, F.	Kisenyi Slum located in Kampala city, Uganda. Kampala city has the highest urban population growth rate in Uganda. Kisenyi slum is the largest informal settlement located in the south-western part of Kampala Central Division. It is a typical urban poor region with the informal settlements, clusters of dilapidated housing, large population size and a rapidly expanding population	Adolescents aged 15–19 years living in Kisenyi slum and implementers of adolescent RH interventions who acted as key informants.<br>Adolescents were purposively identified by community health workers. Followed by criterion sampling by researchers who privately screened each adolescent by asking each one of them secretly (self-report) to stratify them based on sex, schooling status, being sexually active, marital status and ever had a child.<br><br>Total participants= 95 (85 adols - 53 males and 32 females)<br>and 10 key Informants=10 (number for each gender not specified)<br><br>- The first 5 KIs were identified based on the discussion held with the adolescents as they listed key providers in Kisenyi and the rest of the service providers using the snow balling technique by asking the key informants initially interviewed.<br>- Written informed consent was sought from	Assessed the fit between the available interventions and the RH needs of adolescents living in Kisenyi, Uganda	Study was conducted between July 2019 and February 2020<br>Used 8 sessions of FGD for adolescents aged 15–19 years who had lived in Kisenyi for the last 1 year.<br>FGD conducted in local language (Luganda)<br>Interviews for key informants (language not specified).<br>Used conventional content analysis approach.<br>2 community consultative validation meetings with 26 representatives of the adolescents engaged in the initial FGDs.<br>-FGDs and interviews were audio recorded with consent of the participants and then transcribed verbatim. The transcripts were analysed using Atlas Ti version 8.<br>-Ethical approval by the Makerere University School of Public Health Higher Degrees Research and Ethics Committee.<br>Method of confidentiality and anonymity of data specified.<br>- Used RATS (Relevance of study design in answering the research question; Appropriateness of qualitative method;	RH needs of adolescents between 15–19 years living in the urban poor settings of Kisenyi, design of the available RH interventions and the extent to which the design align with the RH needs of these adolescents	This was reported on the following pre-defined themes: Number 3 is a Provider- related theme<br><br>1. Sexual reproductive health and social needs- Adolescents wanted sexual health information, RH services and products and service delivery arrangement with an emphasis on quality and adequacy. Information and RH services and products included the need for: regular sexual health information, quality family planning services, quality STI/HIV/AIDS testing and treatment, adequate supply of quality condoms, antenatal care for young mothers, abortion services and newborn care services.<br>§ Top three prioritized sexual RH needs for the females were quality family planning, quality STI/ HIV/AIDS testing and treatment and accessing affordable (free) general medical services (in that order).<br>§ The males' priority sexual health needs were quality STI/ HIV/AIDS testing and treatment followed by sexual health information.<br>Social needs included need for: livelihood and decent employment, sanitation, parental guidance, substance abuse management in the community, housing, and drug abuse rehabilitation.<br>*"Jobs!...give us jobs that will build us …. When we have nothing, we end up stealing"(Male participants FGD4)*

A

Study reference	Title	Year	Authors	Setting	Sample	Aims	Study Design	Phenomenon Explored	Findings
					adolescents 18–19 years<br>- (Written assent obtained from parents/guardian for adolescent child (15-17yrs)<br>-		Transparency of procedures in sampling respondents and Soundness of interpretive approach as applied to the data analysis) to ensure rigour.		*"The poverty is very high and when you get infected with diseases like Candida you can't even go for treatment because of lack of money … ..We fall sick all of the time, the trenches here spread diseases due to poor sanitation"(Female participant FGD7).*<br>§ Female adolescents prioritized livelihood and sanitation while, the male adolescents prioritised livelihood and housing.<br><br>2. Design features of the available adolescent RH interventions in Kisenyi slum<br>Providers did not offer full range of SRH services to adolescents, used facility-based model and targeted only urban beneficiaries, not specific to urban poor. - design and implementation of their interventions depended on their understanding of the need and their capacity to provide the respective intervention.<br>3. Extent of fit between intervention design features and priority reproductive health needs of the adolescents.<br>o Adolescents reported that Intervention was only in moderate fit with the adolescent needs because of limited focus on social needs like livelihood and sanitation.<br>o Livelihood intervention such as empowering them to improve their negotiation power in relationships which may improve their reproductive health overall.<br>o Adols perceived that poor sanitation resulting from poor toilet facilities affected their RH causing urinary tract infections and poor menstrual hygiene<br>o Adolescents preferred regular holistic community outreaches to facility-based interventions.

A

Study reference	Title	Year	Authors	Setting	Sample	Aims	Study Design	Phenomenon Explored	Findings

A

Study reference	Title	Year	Authors	Setting	Sample	Aims	Study Design	Phenomenon Explored	Findings
03<br><br>(eligible)	What do South African adolescents want in a sexual health service? Evidence from the South African Studies on HIV in Adolescents (SASHA) project	2018	Smith, P., Marcus, R., Bennie, T., Nkala, B., Nchabeleng, M., Latka, M.H., Gray, G., Wallace, M. and Bekker, L.G.	Involved 5 clinical research sites in South Africa:<br>1. the Desmond Tutu HIV Foundation's Emavundleni clinical research site in Crossroads, Cape Town<br>2. the Centre for the AIDS Programme of Research in South Africa in Durban<br>3. the Perinatal HIV Research Unit in Soweto, Johannesburg<br>4. MeCRU Clinical Research Unit at Sefako Makgatho Health Sciences University (the former Medunsa Campus of the University of Limpopo) in Pretoria<br>5. the Aurum Institute in Klerksdorp and Rustenburg.<br>- These research sites are located in predominantly low-income areas with a high HIV incidence.	- Adolescents between 12 and 17 years were recruited from community outreach activities in sports clubs, transport hubs, community centres, HIV voluntary counselling and testing services and other public spaces.<br>- A total of 120 participants (54 male, 60 female, 6 unspecified) across five sites were recruited. Other social demographics not specified.<br>- Method of sampling not specified.	To examine the needs of South African (SA) adolescents with regards to differentiated, accessible and adolescent responsive SRHS	- Qualitative. Trained community educators conducted the FGDs at the research sites.<br>- Concept mapping was used to facilitate discussion of adolescents' opinions because of the sensitiveness of SRH issues.<br>- FGDs were conducted in the local languages, audio recorded and transcribed, the into English.<br>- Nine representatives from the five sites were trained to code the FGDs, develop coding charts and map the data.<br>- Framework analysis was used to organise data into themes.<br>- Ethical approval for the study was granted by each site's institutional review board.<br>- How the issues of confidentiality and anonymity was addressed was not specified.<br>- Method of rigour not specified as well.<br>- written informed consent obtained,	Adolescent preferences for differentiated SRHS.	4 themes emerged:<br>1. The need for the provision of dedicated adolescent health services.<br>-Adolescents rejected ''routine care at government clinic facilities, reporting that clinic opening times were inflexible, that staff could be unpleasant, stigmatising and often rude, and that staff did not respect confidentiality and gave inappropriate and misleading information''<br>-Wanted dedicated and adolescent friendly services from younger staff such that they would experience less discrimination, with reduction in perceived and actual stigma.<br>*"'It's okay if we will not feel shy and become free, if we can be understood by staff. Younger staff can understand challenges facing adolescents and address our issues as adolescents. They must be able to talk to people. One can go to the clinic and find that there are staff that are not kind or sensitive.' (Male, 15 - 17)*<br>2.Tailored services with developmentally appropriate information.<br>-Adolescents wanted tailored information and services directed at their specific developmental stage and needs.<br>-Expressed that staff could encourage healthy behaviour if they were open to listening to young people, could connect with common issues faced by adolescents, could give relevant practical advice, and could suggest ways of navigating potential barriers to health<br>-Didactic and punitive commands issued by clinic staff were specifically noted as being unhelpful.<br>3.Emphasis on the desire for confidentiality and trusting relationships with healthcare staff. Adolescents expressed need for trust in staff and health facilities because

A

Study reference	Title	Year	Authors	Setting	Sample	Aims	Study Design	Phenomenon Explored	Findings
							including consent for audio recordings of the FGD. Authors stated that parental consent was sought for participants aged less than 18years.		public health facilities are places where they may see people they know. They stated that v Participants stated that mistrust leads to less honest sharing of information, but that trusting relationships would foster honest conversation with healthcare professionals.<br>4.Availability of services and information.<br>-Adolescent emphasised need for contraception services and related education, stating that these services should be available and confidential.<br>*"… it is better to get information from experienced adults like your mother or health staff '*<br>-Also preferred information on prevention of sexually transmitted infections and individualised contraception options, instead of receiving a 'one-size-fits-all' approach and suggested that women should have access to private rooms where contraception can be made available.<br>-Participants believed that clinic staff did not always present them with all the information, or all the available options and thus recognised the need for accurate information in making health decisions.

A

Study reference	Title	Year	Authors	Setting	Sample	Aims	Study Design	Phenomenon Explored	Findings
04<br><br>(eligible)	Engaging young people in the design of a sexual reproductive health intervention: Lessons learnt from the Yathu Yathu ("For us, by us") formative study in Zambia	2021	Simuyaba, M., Hensen, B., Phiri, M., Mwansa, C., Mwenge, L., Kabumbu, M., Belemu, S., Shanaube, K., Schaap, A., Floyd, S. and Fidler, S.	2 high-density peri-urban communities in Lusaka, Zambia used. Community names not mentioned. The study communities had limited recreational facilities for AYP, and poverty and unemployment were reportedly high.	- AYP aged 15-24 years were purposively selected based on age, sex, where they lived, and positions held in the study communities.<br>- In total, 215 mixed group AYP (male and female). Total number of males and females not mentioned. Table provided on the demographics not clear because it indicates a total of 251 AYP which is different from the 215 documented in the narrative.<br>- 21 adults also participated in the research but only participated in the community mapping discussions.<br>- Purposive sampling used for recruitment.	To co-design SRH intervention with AYP through participatory qualitative research	- Participatory qualitative methods.<br>- 4 observations in each community through spiral walks at the main government health facility in each community to observe the delivery of services to AYP (Adolescents and young people)<br>- 10 FGDs and 8 IDIs were conducted with AYP across the 2 communities.<br>- Embedded within FGDs and IDIs were participatory activities, including community mapping, concept mapping and ranking.<br>- Sessions were audio recorded and transcribed verbatim.<br>- Reflective notes were documented<br>- Rigour ensured through debriefing and triangulation.<br>- Ethics obtained from the London School of Hygiene and Tropical and the University of Zambia Biomedical Research Ethics committee. Written consent obtained and for AYP aged < 18, parents/guardians provided written	Engaging AYP in finalising the design of comprehensive SRH intervention for AYP aged 15–24.	3 themes emerged:<br>1.Community mapping: context of AYP's SRH.<br>-AYP gathered in communal spaces such as bars, bus stops/stations, markets, sports facilities, schools, and churches, for different activities.<br>-Alcohol and drug abuse, peer pressure and lack of recreational facilities were factors identified as influencing AYP's sexual behaviours.<br>*"In the community it is much better…they [AYP] say that mostly people are not comfortable coming to the clinic [health facility]…they are not comfortable with the workers [HCWs]…some say that they are afraid of meeting people they know," (young woman, 23 years, IDI community 1*<br>*"Sometimes it is difficult or embarrassing to open up to someone of the opposite sex," (20 year old young woman, IDI, community 2).*<br>-Some ABYM were involved in gangs which influenced decisions to access services and participation in crime, alcohol, and drug use.<br>-Adolescents were reluctant to access SRH services because of fear of being reprimanded by health care workers (HCWs) since accessing SRH services is considered culturally inappropriate for AYP by the communities.<br>*"…sometimes you need fellow youths [peers] because we know that although they are testing people [offering HIV testing], they also experience what we experience…they know what they are talking about because they also go through the same things we experience," (Young man, 15–17 years FGD, community 2).*<br>-Not all services were readily available at the local health facilities. For example, voluntary medical male circumcision (VMMC) and cervical

A

Study reference	Title	Year	Authors	Setting	Sample	Aims	Study Design	Phenomenon Explored	Findings
							informed consent. Confidentiality and anonymity ensured. The 2 communities were anonymised as 1 & 2 as part of the anonymity procedures.<br>- Thematic analysis done based on the pre-determined data collection process		cancer screening services were not provided in community.<br>2.Primary discussions.<br>-Adolescents lacked knowledge of some services, including post and pre-exposure prophylaxis for HIV prevention and some contraceptive method.<br>-They also preferred delivery of SRH services in AYP specific spaces such as markets, bus stops, churches, schools, and sports fields, rather than at the health facility. However, services, including VMMC, cervical cancer screening and antenatal care, many AYP considered it important that these be offered at the health facility for reasons related to privacy and the level of expertise required to deliver these services.<br>-Wanted service providers to be welcoming, approachable and have a non-judgemental attitude towards AYP.<br>-Also needed community sensitisation and distribution of prevention point cards (PPC) as proposed by the researchers. The authors view PPC as loyalty cards to gain points for accessing services and redeeming these points for rewards<br>-AYP suggested financial and school support, electronic devices, clothing, and food supplies as rewards<br>3.Consolidation of AYP's views.<br>-From adolescents' views, the authors suggested locations for hubs and services to be offered and design.<br>-Adols suggested that one hub could cater for everyone (adolescent population). HIV testing were preferred compared to oral pre-exposure prophylaxis because they were unfamiliar with the prophylaxis.<br>-They preferred having a PSW located at the health facility to facilitate referrals from the hubs.

A

Study reference	Title	Year	Authors	Setting	Sample	Aims	Study Design	Phenomenon Explored	Findings
									**-For branding, the majority of AYP chose lemon green, orange and white as colours to be used for the PPCs, hubs and other materials. Thus, providing input in the design of the cards.**

A

Study reference	Title	Year	Authors	Setting	Sample	Aims	Study Design	Phenomenon Explored	Findings
**05**<br><br>**(eligible)**	**Youth accessing reproductive health services in Malawi: drivers, barriers, and suggestions from the perspectives of youth and parents.**	**2018**	**Self, A., Chipokosa, S., Misomali, A., Aung, T., Harvey, S.A., Chimchere, M., Chilembwe, J., Park, L., Chalimba, C., Monjeza, E. and Kachale, F.**	**3 out of the 28 districts of Malawi (Dowa, Machinga, and Phalombe) were selected with 2 facilities from each district. Selection was based on the decreasing trend of total fertility rate and age-specific fertility rates of from the 2010 Demographic Health Survey, variation in the non-governmental organizations providing FP services and geographic accessibility**	**- Involved 255 youth (15-24years) and 40 parent/guardian**<br>**- Study intended to also recruit both female and male parents/guardians for the parent FGDs, but no male parents or guardians participated. Parents of female youths were selected due to logistics, but study did not exclude parents of male youths.**<br>**- Among out-of-school males both married and single males participated**<br>**- Purposive sampling used for recruitment.**	**To explore the perspectives of youth and adults about the drivers and barriers to youth accessing family planning in Malawi and their ideas to improve service**	**- Qualitative study used semi-structured focus group discussions (FGD).**<br>**- They conducted all FGDs in Chichewa language using translated guides**<br>**- The Johns Hopkins Bloomberg School of Public Health Institutional Review Board reviewed and approved the study gave approval for the study. Informed consent obtained and assent sought for minors.**<br>**- Focus group discussions had between 5 and 10 participants and took between 50 and 110 min. One moderator and one note-taker.**<br>**- Sessions were audio recorded and transcribed verbatim.**<br>**- All FGDs were translated and transcribed verbatim back into English. A selection of the transcripts was checked further for transcription and translation accuracy**<br>**- Confidentiality and anonymity ensured.**	**Perspectives of youth and adults about the drivers and barriers to youth accessing family planning and their ideas to improve services.**	**Themes (from adolescent population only):**<br>**1.Drivers of youth accessing family planning services.**<br>**-Societal benefits and personal protection emerged as the main motivators for youth to use family planning (FP). Societal benefits included: managing population growth; reducing demand for public services; and reducing population-related adverse effects, such as food and water shortages, environmental degradation, and unsustainable pressure on the government to provide public goods and services.**<br>**-Need for youth to protect themselves and avoid negative consequences from unprotected sex as another motivating factor. Participants mentioned preventing unwanted pregnancies, avoiding birth complications as a result of adolescent pregnancies and improper child spacing; fistulas, not wanting to die at an early age as a result of HIV, and protection from sexually transmitted diseases as drivers for youth accessing FP.**<br>***"If we use contraceptives we can have a manageable number of children, rather than having so many that we can't raise them. Through contraceptives the woman has time to raise her child, and the child can grow healthily." (Female, married, 18–24 yrs., Dowa)***<br>***"No its all the same, family planning is very important to everyone." (Male, out-of-school, 15–20 yrs., Dowa)***<br>***"Girls are the ones who carry the burden of child birth. The man can walk around freely and claim that he has no children, while girls cannot, they have to carry the baby on their back." (Female, out-of-school, 15–18 yrs., Machinga).***<br>**2.Barriers youth face accessing family planning services**

A

Study reference	Title	Year	Authors	Setting	Sample	Aims	Study Design	Phenomenon Explored	Findings
							- **Data was thematically analysed.**		**Misconceptions and perceived side-effects.<br>-Barriers included misconceptions, which were thought to cause permanent sterility, illness, cancer, and weaken men's libido. Participants had the fewest misconceptions about condoms and the most misconceptions about oral contraceptive pills. Male youth and out-of-school youth were more likely than female and in-school youth, and parent participants to talk about contraceptive misconceptions.<br>-Youth expressed a preference for condoms over other contraceptive methods because of the perceived side effects.<br>-Participants also said costs were a barrier to use of contraceptives. Some NGO providers regularly charge for certain services. Most youth said that they prefer government providers over NGO providers largely because they are supposed to be free. However, participants in one district reported that some government providers charged fees.<br>-Both male and female youth mentioned transport costs and long distances as another barrier to seeking FP services.<br>-Many youths wanted FP services closer in their communities because of the distances and transport costs, while other youth were okay with services being further away since the distance protected their privacy.<br>3.Suggestions from participants for improving family planning services.<br>-The most common suggestion among youth participants was the formulation of youth clubs, where youth could share FP information and health providers could offer FP counselling, education, and commodities.<br>-The second most common suggestion among youth participants**

A

Study reference	Title	Year	Authors	Setting	Sample	Aims	Study Design	Phenomenon Explored	Findings
									**and the most common among parents was the need for more FP counselling for youth. Participants said this counselling would ensure youth understand the importance of FP and how methods work.**

A

Study reference	Title	Year	Authors	Setting	Sample	Aims	Study Design	Phenomenon Explored	Findings
06<br><br>(eligible)	Young people's perceptions of youth-oriented health services in urban Soweto, South Africa: a qualitative investigation.	2014	Schriver, B., Meagley, K., Norris, S., Geary, R. and Stein, A.D.	Soweto in South Africa, an urban township situated to the southwest of Johannesburg.<br>Reason for the choice of setting not stated	Involved 23 Black African young men and women from a subset of fifty named ''BT20 cohort members'' selected using purposive sampling. This subset was randomly selected from a full cohort to participate in a pilot of the periodic Young Adolescent Health Survey (YAHS).<br>- The selection of the sub-set was based on gender (14 female/9 male) and utilisation of health services within the last six months (15 users/8 non-users).<br>- According to the authors, the BT20 cohort is the largest and longest running study of child and adolescent development on the African continent. This cohort been studied regularly for over 20years.<br>- All participants were aged 21 or 22 at the time of the interview.	To examine the knowledge and perceptions of current health services oriented towards young people and examine potential alternative approaches to health service delivery	-Used grounded theory as described by Borgatti and Strauss and Corbin<br>-25 in-depth interviews were conducted between May-July 2012. However, 23 (14 female, 9 male) samples were used for data analysis. The 2 pilot interviews were not used because as they did not reflect the overall data collection methodology used.<br>-Ethics approval was obtained by the University of Witwatersrand under the BT20 approval (ID M120138).<br>-Participants were contacted by phone and invited them to be interviewed. Informed consent obtained.<br>-All interviews were in English.<br>-Interviews varied in length between 45 minutes and two hours and were recorded and transcribed verbatim.<br>-2 primary investigators independently read all transcripts and developed codebooks based on emerging themes.<br>-Inter-coder agreement was checked for three interviews; discrepancies in coding were discussed and a consensus was reached.<br>-Data analysed using MAXQDA10 Qualitative Analysis Software.	Perceptions of youth-oriented health services	3 themes emerged:<br>1.Perception of current health services.<br>-Young people were dissatisfied with the current public health services in Soweto. Dissatisfaction was linked to a lack of resources, long waiting times, and poor quality of care.<br>-Staffing shortages, insufficient diagnostic equipment (such as xrays) and drug stock-outs were reported. Because of frequent drug stock-outs, clinics often only offered basic medications such as antibiotics or generic painkillers like Panadol which are readily available at small shops and supermarkets. Participants felt that going to a clinic was an unnecessary step that did not always result in better outcomes.<br>2.Knowledge of YFS initiatives.<br>-knowledge of YFS programmes was very low. From the 23 participants interviewed only three reported ever hearing about 'youth-friendly services,' no participant was able to express extensive knowledge of the programme's purpose or activities.<br>3.Attitudes toward alternative health services.<br>-On participant's opinions of the two alternative health service delivery systems: School Based Health Clinics (SBHC) and Community Health Workers (CHW). There was no service that was preferred. A few participants seemed sceptical about the likelihood of these services being implemented in Soweto - potentially reflecting their feeling of disenfranchisement with current health service interventions.<br>-Health education and HIV testing and treatment were among the services participants would most like to see offered by CHWs. Other services that were discussed included general check-ups, health education and assessments for

A

Study reference	Title	Year	Authors	Setting	Sample	Aims	Study Design	Phenomenon Explored	Findings
									**obesity, blood pressure, diabetes, and illness**

A

Study reference	Title	Year	Authors	Setting	Sample	Aims	Study Design	Phenomenon Explored	Findings
**07**<br><br>**(eligible)**	**Provision of Reproductive Health Services for Adolescents -- Report of a Study in Two Local Government Areas (LGAs) of Nigeria.**	**1996**	**Olukoya, A**	**-Involved 2 LGAs in 2 states of Nigeria. Barkin Ladi Local Government Area (LGA) in Plateau State in northern Nigeria, and Ijebu-Ode LGA in Ogun State in the south-western Nigeria.**<br>**-These sites are WHO focus LGAs and represent a northern and a southern location, respectively.**<br>**-Urban and rural locations from each LGA were selected. A General Hospital which provided comprehensive services, Primary Health Care Centres, as well as School Health Clinics were chosen, in both urban and rural locations of the LGA where available.**<br>**-The health facilities were chosen based (apart from urban/rural representation) on perceived volume of use of services by adolescents, choosing facilities perceived to be more patronised by adolescents, or more likely to be so patronised**	**-6 out of school adolescents and 2 in school adolescents, 1 community leader and 1 health worker. Age of adolescents unclear as authors mentioned less and greater than 15 years. Total adolescents=8.**<br>**-Also, IDIs with Medical Officer of Health (where available), key nursing personnel, and head of referral facility. However, the number of participants from IDIs not documented.**	**To assess the provision of reproductive health services for adolescents in two Local Government Areas of Nigeria.**	**-Mixed study**<br>**-Data collection methods included direct observation over a period of time not mentioned, FGDs (20), Use of exiting records from October 1992 to October 1993 from selected facilities in both LGAs onto pre-designed forms.**<br>**-Unclear how many adolescents participated in the 20 FGDs**<br>**-documentation on ethical consideration and rigour of the data collection process and method of data analysis as well.**<br>**-Unclear what language was used for data collection. Documentation not clear on transcriptions, coding, and emerging themes.**	**Provision of Reproductive Health Services for Adolescents**	**Discussed under the following headings without mentioning whether they emerged as themes from the FGD and interview transcripts**<br>**1.Perceptions of the health problems of adolescents.**<br>**-Adolescents) perceived health problems in general terms as they mentioned lack of potable water, poverty, poor sanitation as priority problems. Abortion and gonorrhoea were mentioned and perceived as serious problems affecting young people. Other health problems mentioned included sickle cell disease (the northern group), delinquency, drug abuse and alcoholism.**<br>**-Adolescents believed that early marriage was bad for the adolescent because it could lead to poverty and inability on the part of the two people involved to progress well in life. They said it leads to financial problems which often leads to divorce. Other bad effects mentioned is malnutrition and poor "up-bringing" in the offspring of such couples.**<br>**-Many of the adolescents believed that their risk of contracting STIs including HIV/AIDS was high because of their sexual activities.**<br><br>**2.Help-seeking behaviour**<br>**-Adolescents said they would go to the hospital for perceived illness. That is after trying first aid especially at the chemists as well as sometimes.**<br>**-Most felt that the health facilities are not enough, and that the equipment and drugs are usually not available. Some in-school adolescents felt that health care providers were not always nice, and that they do not refer serious cases in time.**

A

Study reference	Title	Year	Authors	Setting	Sample	Aims	Study Design	Phenomenon Explored	Findings
									**-Did not perceive the health care services as sources of information on sexuality problems. Preferred friends and senior siblings.**

A

Study reference	Title	Year	Authors	Setting	Sample	Aims	Study Design	Phenomenon Explored	Findings
08 (eligible)	Sexual and reproductive health services (SRHS) for adolescents in Enugu state, Nigeria: a mixed methods approach.	2018	Odo, A.N., Samuel, E.S., Nwagu, E.N., Nnamani, P.O. and Atama, C.S.	-Conducted in Enugu State, Southeast Nigeria between January 2015, and July 2016. The state comprised 17 Local Government Areas (LGAs) with an estimated total population of 3,267,837.<br>-Some of these LGAs have commercial areas like big markets and hotels that attract visitors who come for one business or the other.<br>-Adolescents in these areas unlike those in non-commercial areas, engage in business such as hawking and commercial sex working. Their males also engage in commercial motor-cycle riding (Okada) which exposes them to rough or unhealthy lifestyles.	Qualitatively:<br>-6–10 males and 6–10 female adolescents aged 12-22 years were recruited from the questionnaire respondents. This gave 2 groups (1 male group and 1 female group) from each LGA, giving a total of 18 focus groups. No mention of the total number of adolescents who participated in the study.<br>-Convenience sampling technique was also used to select 3 (1 male and 2 female) interviewees from each LGA for IDIs. This gave a total of 27 interviewees. Not clear on the total number of adolescents that participated in the study	To determine the availability and accessibility (geographical and financial) of sexual and reproductive health services (SRHS) among adolescents in Enugu State, Nigeria	-A mixed method (quantitative and qualitative) approach was employed.<br>-Interviews (type not mentioned) and FGD were used for the qualitative while checklist and questionnaire constituted the quantitative methods.<br>-Convenience sampling technique was used to select 6–10 male and 6–10 female adolescents.<br>-The discussions and interviews were recorded with digital tape recorders. Non-verbal cues were recorded by note taking.<br>-Although, unclear what language was used for data collection, discussions and interviews were transcribed in English language.<br>-Ethical approval was obtained, and informed consent sought. However, no documentation on rigour of the data collection process.<br>-The NVivo 11 Pro software was used to code and analyse the data thematically	The availability and accessibility (geographical and financial) of sexual and reproductive health services (SRHS).	Qualitative findings<br>1.Availability of SRHS for adolescents<br>-Adolescents reported that some of the SRHS were available but not particularly for adolescents. The available services for adolescents reported were sexuality education which were provided in church and the secondary schools, through other health related subjects, and services for prevention and management of STIs and HIV and AIDS, which were mainly provided by churches and schools during youth week.<br>*"I get sexuality education services and services for prevention and management of STIs and HIV and AIDS in the school and church during youth's week but for others, I don't know about them" (Udenu 002).*<br>*"I have not received such (family planning services), and I don't think it is made for adolescents. It is only for married couples" (Enugu-North 003)*<br>2.Accessibility of SRHS to adolescents<br>-Adolescents reported that they have geographical access to public health facilities that provide general SHRS, as they indicated that they can walk to the health facility within 30 minutes; except in rural areas where some accessible health facilities do not provide some of the SRHS and are not affordable.

A

Study reference	Title	Year	Authors	Setting	Sample	Aims	Study Design	Phenomenon Explored	Findings
**09**<br><br>**(eligible)**	**Access to information and use of adolescent sexual reproductive health services: Qualitative exploration of barriers and facilitators in Kisumu and Kakamega, Kenya**	**2020**	**Mutea, L., Ontiri, S., Kadiri, F., Michielesen, K. and Gichangi, P.**	**-2 wards: Kobura ward within Nyando sub-county (Kisumu county) and Kholera ward within Matungu sub-county (Kakamega County). These two wards were purposively selected because they are among those with the highest burden of adolescent pregnancy and low use of ASRH services within the two counties.**	**-Male and female adolescents aged 15-19 years. Total =70 (33 females; 37 males).**<br>**-Community representative = 12 (2 females; 10 males)**<br>**-Teachers = 14 (11 females; 3males).**<br>**-Health Care Workers = 10 (7 female; 3 males).**	**To describe the barriers to and facilitators of access to adolescent sexual and reproductive health services in Kisumu and Kakamega counties, Kenya**	**-Qualitative mentioned.**<br>**-Study enrolment was done through catchment health facilities, surrounding schools, and the community within the participants' health facility catchment area—the surrounding geographic area from which a health facility attracts its clients.**<br>**-Consent and ethics obtained from the Kenya Medical Research Institute research ethics reviews committee and the Johns Hopkins School of Public Health Institutional Review Board.**<br>**-FGD, IDIs and Key Informant interviews (KIIs) used.**<br>**-All FGDs; IDIs and KII were conducted in English except the IDIs with community representatives, which were conducted in Swahili, Lwanga, and Dholuo and transcribed subsequently to English for analysis.**<br>**-Transcripts were analysed with the use of NVivo 12 software.**<br>**-Thematic analysis done.**<br>**-Evidence of qualitative rigour during data collection and analysis.**<br>**-Used ecological model to show the relationship between the study variables.**	**Barriers and facilitators to access and use of SRH services by adolescents**	**Themes: (from youth population only)**<br>**1.Common issues perceived to affect the health of adolescents in the Community**<br>**-Adolescents reported facing issues such as lack of access to contraceptives services and methods, particularly condoms.**<br>**-Participants mentioned child marriage was a challenge; because of poverty, parents offered their daughters for marriage in exchange for dowry.**<br>**-Adolescents, largely male also reported using bhang (marijuana) and alcohol due to pressure and social problems at home.**<br>***"When they are peer pressured, they can take alcohol and that can lead to drop out of school." (Kisumu, FGD 1, Girls)***<br>***"[One of the] things that causes rapes are the use of drugs. You may find that even an old grandmother may be raped by young people because drugs have destroyed their brains. Drugs use may also cause raping a child who is underage." (Kisumu, FGD 1, Boys***<br>***"You can get a young girl being convinced by an older person who has got money to indulge them in sex." (Kakamega, FGD 1, Girls***<br>**2.Barriers to access and use of SRH information and services by adolescents.**<br>**This was reported at all five levels of the ecological model: individual, relationship, organization, community, and policy.**<br>**-Individual-level barriers: The most common barrier among adolescent participants that mentioned in all FGDs and IDIs was the lack of money to access services, including for transport to the health facility, consultation, and medicine fee.**<br>***"When someone wants to go to the hospital, they may lack the fare to***

A

Study reference	Title	Year	Authors	Setting	Sample	Aims	Study Design	Phenomenon Explored	Findings
									take them there, or say it's at night, getting a vehicle that takes them may be hard." (Kakamega, FGD 1, Girls<br>***"Those who are in adolescence choose places where they are not known so that when they are diagnosed with certain illness, it will be a secret, and no one will know." (Kisumu, FGD 1, Girls) Maybe when you go to the hospital, you know that somebody from your clan is there. So, you fear going there because they might find out and tell your parents." (Kakamega, FGD 1, Boys and Girls***<br>**-Relationship-level barriers: Some respondents reported that the absence of parental teachings on ASRH discouraged use of care services. SRH matters were rarely discussed at home; culturally, SRH is not a topic discussed much because adolescents are perceived as children and not as young adults with an active sexual life.**<br>***"I have parents at home, but maybe I'm afraid to talk to them. So, if someone else's parent is discussing it, I could ask questions and they are ready to answer. Asking my father, he might have trouble talking to me about such things." (Kakamega, IDI, Boy 3) "Parents do not want to give their children time to access this information, maybe because they feel it is not the right time. . ., but generally somebody who is 15 years, that one to me needs lots of counseling and guidance from both home and outside home." (Kisumu, IDI, Community Representative 4)***<br>**-Organization-level barriers: Were related to health facilities and schools; a long distance to SRH services, inflexible timings of SRH services, shortage of staff and long queues, health facility costs, and supply stock-outs were reported as**

A

Study reference	Title	Year	Authors	Setting	Sample	Aims	Study Design	Phenomenon Explored	Findings
									**organizational barriers to use of SRH care services.**<br>***"People might overhear. . . I felt that the place was not very private. Getting out of there was a big problem because I felt like everyone had heard what we talked about with the doctor." (Kakamega, IDI, Boy 3) ". . . the doctor reads your name out loudly in public and what you are suffering from. This may make you even leave and go back home." (Kisumu, FGD 1, Boys***<br>***"The number of people in the facility can make you stay there until evening." (Kakamega, FGD 1, Boys***<br>***In this community I have not seen [youth-friendly spaces] because when you go to the hospitals, they just treat you like any regular patient." (Kisumu, FGD 2, Boys)***<br>***"Sometimes you got to the facility and the tell you that they don't have medication or oral syringes." (Kakamega, IDI, Boy 3)***<br>**-Community-level barriers. Social stigma was reported as a barrier because adolescents did not want anyone, they knew to see them at the health facility receiving SRH services due to association of ASRH services with sexual activities, which were sometimes labelled as "bad manners." Religious beliefs about adolescent SRH were a barrier to access to services, with many Christian leaders expecting adolescents to abstain from sex.**<br>***"Stigma, if you are seen going to the hospital, it's like you're engaging in sex. So, society will have a particular perception of you." (Kisumu, IDI, County leader 1)***<br>***"They [community members] will just see her and say, 'She involved herself in unsafe sex, that is why she is pregnant.' Could be maybe she is also exposed to other infections." (Kisumu, IDI, Girl 3***

A

Study reference	Title	Year	Authors	Setting	Sample	Aims	Study Design	Phenomenon Explored	Findings
									**-Policy level barriers: Participants mentioned lack of knowledge of ASRH policy as a barrier to ASRH services. This meant that health service providers and teachers were not fully aware of what information and services adolescents were entitled to. Implementation of ASRH policies was lacking in some areas. In addition, the education sector had limitation on health workers' engagement with adolescents in school settings.**<br>**3.Facilitators to access and use of ASRH information and services**<br>**-Relationship-level facilitators. Included supportive attitudes by some health workers in some facilities enabled use of ASRH services. A few adolescent females reported positive experiences with the friendly providers who paid attention to the client-specific needs.** ***Parents do not want to give their children time to access this information, maybe because they feel it is not the right time. . ., but generally somebody who is 15 years, that one to me needs lots of counseling and guidance from both home and outside home." (Kisumu, IDI, Community Representative 4)***<br>**-Organizational-level facilitators: At the health facility, adolescent-reported need for motivators including short distance to the health facility, availability of free services and supplies, and privacy during service delivery. Some adolescents appreciated being given priority in the service queues.**<br>**-Adolescents needed school re-entry policy that allows pregnant girls to continue with their studies until they are due to deliver and are readmitted after giving birth.**

A

Study reference	Title	Year	Authors	Setting	Sample	Aims	Study Design	Phenomenon Explored	Findings
**010 (eligible)**	**Does Making Clinic-based Reproductive Health Services More Youth-friendly Increase Service Use by Adolescents? Evidence From Lusaka, Zambia**	**2003**	**Mmari, K.N. and Magnani, R.J.**	**-10 public clinics and their surrounding "catchment" areas in the Lusaka, Zambia metropolitan area were selected. 8 of these clinics offered youth friendly services in connection with the youth friendly service projects while 2 clinics offered only standard reproductive health services, without the additional youth-friendly components.<br>-The clinics were choosen for participation in the various YFS projects in consultation with Lusaka District Health Management Team (LDHMT). Also, they were more predisposed to providing youth-friendly services than the clinics not included in any of the YFS projects.**	**-260 (60 youths who had been to the facility and 200 youths who had never been to the facility) aged 15-24 years<br>-10 managers<br>-30 nurses<br>-20 staff**	**The study evaluated the impact of three youth-friendly service (YFS) projects in Lusaka, Zambia**	**-Mixed (Qualitative and quantitative).<br>-Data collection was carried out by Lemba Associates, a Lusaka-based research firm.<br>-Convenient sampling was used to select the youths that participated in the study<br>-FGDs and interviews were conducted for youths had never attended the health facility and those who had been to the facility (Client exit interview), respectively.<br>-Only IDIs were conducted for managers, nurses, and staff. Unclear whether interviews were conducted for youths in English.<br>-Interviews were conducted in both English and Nyanga languages, tape-recorded, and transcribed.<br>-Unclear how issues of ethics, consent, confidentiality, and anonymity were addressed.<br>-Focus groups took place in schools and outside clinic premises.<br>-Five youth interviewers were trained to interview both male and female clients at each clinic**	**Impact of youth-friendly service (YFS) projects**	**From youth population only:<br>Youth-Friendliness of Health Service (the only theme)<br>-Youths from the non-YFS clinics reported, being ignored when they tried to ask nurses questions about contraceptives. They were told by some nurses that they were "too young" to receive reproductive health services.<br>-Youth also reported that they did not feel their sessions were private/confidentiality because they believed that staff workers like the cashiers, receptionists, and medical clerks could not be trusted to maintain their confidentiality.<br>-Females' youths believed that the nurses would do a good job attending to them because they are trained" and "understand a lot about reproductive health." Males also believed nurses would do a good job, particularly "if they worked closely with the peer educators.**

A

Study reference	Title	Year	Authors	Setting	Sample	Aims	Study Design	Phenomenon Explored	Findings
**011**	**Adolescents living with HIV in the Copperbelt Province of Zambia: Their reproductive health needs and experiences**	**2018**	**McCarraher, D.R., Packer, C., Mercer, S., Dennis, A., Banda, H., Nyambe, N., Stalter, R.M., Mwansa, J.K., Katayamoyo, P. and Denison, J.A.**	**-3 HIV clinics in the Copperbelt Province: 1 children's hospital (Clinic 1) and 2 central hospitals (Clinics 2 and 3).<br>-All clinics offered adolescent-specific clinic days and two provided monthly adolescent support group sessions**	**-32 ALHIV from two HIV clinics (16 male and 16 female ALHIV (ages 15–18); 23 caregivers, and 10 clinic staff who were on ART, their caregivers, and all clinic staff in the children's hospital and in one of the central hospitals**	**Understanding the reproductive health needs of ALHIV and to assess the extent to which these needs are being met.**	**-Mixed study.<br>-Study approved by FHI 360's Protection of Human Subjects Committee, the Eres Converge Institutional Review Board in Zambia, and the Zambia Ministry of Health.<br>-Parental consent sought for ALHIV between the ages of 15 and 17.<br>-Interviews were conducted in either Bemba, Nyanja, or English, and were audio recorded.<br>-Written consent obtained. Not clear on how confidentiality and anonymity was addressed.<br>-IDIs used and were transcribed verbatim and translated (when necessary) into English, reviewed for accuracy, and entered NVivo v.8.<br>-To ensure rigour, 15% of the transcripts were coded by at least two of the authors and discrepancies resolved through discussion'<br>-Thematic analysis done.**	**Reproductive health needs of ALHIV**	**From youth population only:<br>-experiences including forced sex<br>-Adolescents reported that the forced sex was their only experience with sex.<br>-participants reported that perpetrators were family members (step-father, uncle, sister-in-law), and a male friend.<br>-Participant stated that they were afraid that disclosing the violence would lead to them being evicted from the home.<br>-Adolescents reported that sexual experiences stemmed from sexual abuse and felt that they needed to be educated to avoid situations that make them vulnerable to violence.<br>2.Sex partner age and HIV disclosure<br>-Adolescents reported that HIV disclosure to sex partners was rare, mostly because of fear of gossip or rejection by partners.<br>3.Contraceptive use<br>-Reasons for not using contraception, included having only infrequent or spontaneous sex.<br>-Some youth had misperceptions about pregnancy and pregnancy prevention<br>4.Fertility desires and prevention of mother-to-child transmission of HIV.<br>-Adolescents reported they wanted children in the future. Some reported they wanted to wait to have children until after they finished school, after they were married, when they had a job, and/or when they had income. Need for HIV-negative baby was expressed.**

A

Study reference	Title	Year	Authors	Setting	Sample	Aims	Study Design	Phenomenon Explored	Findings
012 (eligible)	Rights-based services for adolescents living with HIV: adolescent self-efficacy and implications for health systems in Zambia	2013	Mburu, G., Hodgson, I., Teltschik, A., Ram, M., Haamujompa, C., Bajpai, D. and Mutali	-1 rural (Kalomo) and 2 urban districts (Lusaka and Kitwe) in Zambia, where adolescents make up almost a quarter of the population of 10 million.<br>-These districts were selected for variation in geographic location, contexts, and interventions available.<br>-In all three districts, adolescents were accessing clinical HIV services from government HIV clinics and non-clinical support from community centres run by non-governmental organisations which served as our study recruitment sites,	-Involved 58 adolescents living with HIV aged 10 - 19years.<br>-14 health care providers (5 doctors, 4 nurses, 2 counsellors, 2 clinical officers and 1 administrative staff	To document the psychosocial and sexual and reproductive health needs of adolescents living with HIV in Zambia	-Qualitative. No specific design mentioned.<br>-Study conducted between April and December 2010<br>-Used Semi-structured interviews and FGDs which were used.<br>-Interviews were conducted in English or local languages (Bemba, Lozi and Nyanja), and audio recorded, transcribed, and translated into English.<br>-Eight focus group discussions were subsequently held with 53 additional adolescents living with HIV, two with 21 parents (1 urban and 1 rural) and three with 24 health care providers (2 urban and 1 rural). Semi-structured discussions done using open-ended questions, conducted in English lasting lasted about 50–60 minutes.<br>-Nature of recruitment reflected purposive sampling though not mentioned.<br>-Ethical approval was granted by the Biomedical Research Ethics Committee of the University of Zambia.<br>-Parents and guardians signed consent forms for orally consenting adolescents aged 10–18, while adolescents aged 18–19 signed their own consent forms.<br>-Confidentiality ensured.<br>-Interviews and FGDs were digitally recorded, transcribed, and translated to English, and analysed using NVivo 7.	Psychosocial and sexual and reproductive health needs of adolescents	From youth population only:<br>1.A sense of rights, entitlement, and expectation<br>Adolescents seemed to embrace the concept of living positively and the rights of young people living with HIV to enjoy a fulfilling sexual life.<br>*"Everyone was looking at me in a funny way and whispering, 'This is the one who is sick, she has AIDS.' This was very painful for me because I thought that it was my right to disclose to people about my status. I felt that my rights were violated." (Focus group discussion, 16-year-old female, Lusaka)*<br>A majority believed that people living with HIV should be allowed to marry and have children.<br>*""People living with HIV have feelings… [they] are human beings with normal lives, and marriage is a right to everyone, whether HIV positive or not." (Interview, 19-year-old female, Lusaka*<br>*""People living with HIV have feelings… [they] are human beings with normal lives, and marriage is a right to everyone, whether HIV positive or not." (Interview, 19-year-old female, Lusaka*<br>-Adolescents were also articulate about their entitlement to control disclosure of their HIV status.<br>2.Expressing unmet need<br>-Adolescents expressed the need for health providers to respect their privacy, be more friendly and less judgemental.<br>*"I have tried to ask them for more information but they did not give me enough. What they told me was not very useful. I would love to have more access to information and life skills. I do not have enough information about family planning, which is my right." (Interview, 17-year-old female, Lusaka)*<br>*I want to know if I can have a boyfriend because every time I ask*

A

Study reference	Title	Year	Authors	Setting	Sample	Aims	Study Design	Phenomenon Explored	Findings
							-Transcripts were coded inductively to identify emerging themes.		***my mum she tells me not to. I would also love to learn about safe sex and safe motherhood." (Focus group discussion, 14-year-old female, Lusaka)***<br>**-Narratives of rights and entitlements emerged as they accused service providers of failing to meet their expectations.**<br>**-Some expressed the need for faster services and their dislike of long queues, which calls for improved systems and better staffing. This sentiment seemed to be particularly related to the stigma that adolescents were experiencing at HIV clinics, since it was widely assumed that they had acquired HIV through sexual exposure, while society expected them to be abstaining from sex.**<br>**adolescents expected to have access to livelihood activities and skills training such as vocational training e.g tailoring.**<br>***"One thing that I would really like is being taught life skills because as it is, no one teaches us these things. I would like to learn how to cook, and also tailoring." (Focus group discussion, 14-year-old female, Lusaka)***

A

Study reference	Title	Year	Authors	Setting	Sample	Aims	Study Design	Phenomenon Explored	Findings
013<br><br>(eligible)	Accessing Sexual and Reproductive Health Information and Services: A Mixed Methods Study of Young Women's Needs and Experiences in Soweto, South Africa	2015	Lince-Deroche, N., Hargey, A., Holt, K. and Shochet, T.,	-3 clinics from Soweto, South Africa because of the clinics offered reproductive health services. A shopping mall was selected because of its proximity to the three clinics and its popularity among youth.<br>-The mix of recruitment locations according to the authors allowed for different perspectives: those of young women who were seeking public sector clinic services and those living in the participating clinics' catchment areas but not seeking services at the time of recruitment.<br>-At the clinics, young women were recruited from the antenatal clinics, abortion (or termination of pregnancy (TOP) clinics, family planning, and HIV-related service departments.	-Involved 15 young woman aged 18-24, living permanently in Soweto, and speaking English, Zulu or Sesotho were recruited qualitatively.	To assess young women's SRH knowledge and experiences and to determine how they get SRH information and services in Soweto, South Africa	-Mixed study (Only qualitative design considered here)<br>-Grounded theory employed.<br>-Interviews were conducted.<br>-Study was approved by Allendale Investigational Review Board and the Human Research Ethics Committee at the University of the Witwatersrand. Permission also obtained from the Gauteng Provincial Department of Health and the City of Johannesburg.<br>- All participants provided written informed consent.<br>-Authors recruited from shopping mall and three large, public, primary health care clinics.<br>-Women participating in the qualitative interviews were not the same as those who participated in the quantitative aspect.<br>-Interviews were conducted in English, Zulu or Sesotho and lasted 1 hour.<br>-Confidentiality maintained.<br>-ATLAS.ti was used to facilitate coding, sorting, and data management.<br>-Data thematically analysed.	Young women's SRH knowledge and experiences on SRH information and services	1.Contraception: knowledge and access to services<br>-Adolescents expressed knowledge of information on contraception which they claimed was available mainly at clinics, but also including friends and family.<br>*"Yes, they can [get information on SRH]. We get newspapers, magazines, and we learn from the TV, it's just that we ignore these things, but yes, they are informed." [P8::49]*<br>*"Yeah, sisters educate about abortion and they even tell you that abortion is no easy matter because you are then between life and death. They actually teach us about many things." [P1:175]*<br>*"When we ask our parents they become aggressive, they shout at us not wanting to talk to us, saying, 'why do want to know about such things,' …sometimes we go to clinics and then they say 'why do you want to know, you are still too young, why don't you ask your parents,' we say, 'they also don't want to tell us.'" [P8:101]*<br>-Some specifically mentioned that family or friends had given them information or encouraged them to "go for prevention."<br>-Nurses' attitudes and busy schedules were reported as obstacles to getting a contraceptive method.<br>*"When I come here [to the clinic], they ask, "Lady, have you come here to ask questions or to say what is wrong with you?*<br>*" They don't have time, so there is not asking them many questions because when you ask them, they ask, "Did you come for that or are you sick? What is it, lady? There are sick people here, who are in pain. Hurry and say what you want." You see." [P8:269*<br>*"…obviously a person would think to themselves: 'I won't go to the clinic*

A

Study reference	Title	Year	Authors	Setting	Sample	Aims	Study Design	Phenomenon Explored	Findings
									and shame myself' or they just do wrong things on their own: 'let me take pills and help myself, maybe it will help me', she does not want to go to the clinic because she fears the eyes of other people, she wants stuff that will keep her concealed… people should not see me like this, best I do this, nobody will see me doing like this, only I will know. [P3:141]<br>**-Needed challenges in communicating with health care workers be removed to be able to attend to their SRH concerns.**<br>**2.Abortion services**<br>**-Young women reported not wanting to attempt to access abortion services due to safety concerns.**<br>**-Young women spontaneously expressed feeling that abortion was stigmatized. and felt that abortion was generally bad or unsafe.**<br>**-Several reported hearing of someone who died because of abortion, and a few said that they had been told by health practitioners (i.e., nurses, social workers) that abortion was a life-threatening procedure.**<br>**-Some mentioned that for confidentiality reasons some women go to other, illegal, places or even try to abort on their own.**<br>**3.HIV testing and condom use**<br>**-Young women expressed that information on HIV and HIV-related services were available in their communities.**<br>**-They commented that young people can get information on HIV if they are in school, by listening to the radio or watching television, or if they visited a clinic and spoke to a nurse or doctor.**<br>**-Services such as testing and treatment, condoms were available at local clinics, but young people rarely use them because of stigma and fear of being ridiculed.**<br>**4.Gender-based violence**

A

Study reference	Title	Year	Authors	Setting	Sample	Aims	Study Design	Phenomenon Explored	Findings
									-Several young women said that they had been forced to have sex by a partner who wanted to impregnate them before they broke up, either because they had cheated, or because their partner suspected that they had cheated.<br>-Reported partners threatening them or making them feel scared, or hitting, kicking, or punching them.<br>5.Concerns and support systems<br>-Young women expressed not having enough support from the community, clinics, or parents.<br>-They talked about community members gossiping and mocking instead of supporting them.<br>-Health care providers not giving the information that young women need because they are overworked, and parents not discussing SRH with their children.<br>-Felt more comfortable talking to friends and family than partners or boyfriends about their concerns.

A

Study reference	Title	Year	Authors	Setting	Sample	Aims	Study Design	Phenomenon Explored	Findings
**014**<br><br>**(eligible)**	**Adolescents' Reproductive Health Problems, Service Preferences, and Accessibility**	**2017**	**Kimo, K. and Makuria, K.**	**-Conducted in a youth centre in Adama, Ethiopia.**<br>**-Adama is one of the biggest cities in Oromia region where no prior exploration has been done regarding adolescent RH problems and their service preference and accessibility.**<br>**-There are three youth centres in Adama city (two belong to the government; one is managed by local NGO) offering youth related activities such as library service, sports activities, establishment of youth clubs, and RH trainings. The authors preferred the youth centre under local NGO because it provides RH services to many adolescents.**	**The authors documented the following:**<br>**-that samples of FGD constituted adolescents (12-19years) who visited the youth centres for RH services.**<br>**-No mention of the total number of adolescents or health care workers that participated in the FGDs.**	**To assess adolescents' reproductive health problems, service preference, and accessibility in Adama city, Ethiopia.**	**-Mixed study (Only qualitative design considered here)**<br>**-3 FGDs were conducted each consisting of 8-10 participants, while two FGDs were conducted with exclusive homogenous groups (of male and female adolescents). Participants of third FGD included service providers (nurses, counsellors) rendering RH service for adolescents, experts from health centres and health office, and NGOs that work on adolescents and youth programs**<br>**-Local permission was granted from the youth centre**<br>**-Informed consent obtained and confidentiality and anonymity maintained.**<br>**-scanty. None on method of recruitment, data collection process and analysis**	**Adolescents' reproductive health problems, service preference, and accessibility**	**1.Reproductive health service problems and preference (assessed quantitatively).**<br>**2.Reproductive health service accessibility**<br>**-Male adolescents reported that health institutions were visited for condoms and treatment for STDs, while female adolescents indicated that health institutions were visited for unwanted pregnancy and contraceptives.**<br>**-Wanted condoms to be made available in in recreational areas, meeting places, schools, public offices, Kebele associations, and bus stations, in addition, to the usual distribution areas."**<br>**-It was reported that among the existing youth centres, only Family Guidance Association of Ethiopia' clinic's youth centre was providing RH services for adolescents. Other centres were not equipped with the necessary materials and the professionals who provided the services.**<br>**SRH information were preferred from friends.**<br>***'My friend is my confidante.' (Participant 11, 19-year-old female)***<br>***'We are a close-knit family that support each other through everything.' (Participant 4, 18-year-old male)***<br>**-Adolescents needed the establishment of more youth centres, and the activities of existing youth centres could also be expanded. For instance, the existing government youth centres should be strengthened. Mostly men were utilizing RH service more than females. Therefore, female should be encouraged to reach at these service areas**<br>**-Adolescents desired to have the required service any time they desired to have it.**

A

Study reference	Title	Year	Authors	Setting	Sample	Aims	Study Design	Phenomenon Explored	Findings
**015**	**Living as an adolescent with HIV in Zambia – lived experiences, sexual health, and reproductive needs.**	**2012**	**Ian Hodgson, Julia Ross, Choolwe Haamujompa & D. Gitau-Mburu**	**The study was conducted in two urban (Lusaka, Kitwe) and one rural (Kalomo) Zambian sites. The settings according to the authors were selected to ensure broad representation of service delivery settings.**	**-Samples consisted of 111 adolescents (10-19years) recruited from ART clinics and other HIV centres run by non-governmental organisations; 38 Key informants, including health care providers (medical, nursing and counselling staff) and 21 parents/guardians were included to allow triangulation.<br>-Participating adolescents were accessing HIV services and aware of their status<br>-Participants were recruited based on their interest and availability<br>-The authors stated that there was no attempt to ensure age or gender homogeneity of participants.<br>-Sample size was pre-defined based on a pilot study.**	**To explore and document the informational, psychosocial, sexual and reproductive health (SRH)needs of adolescents living with HIV in Zambia and identify gaps between these needs and existing services.**	**- Qualitative<br>-Used semi-structured interviews and focus group discussions (72 interviews and 14 FGDs were conducted).<br>-Purposive sampling used for recruitment<br>-Data were collected between April and December 2010.<br>-The Biomedical Research Ethics Committee of the University of Zambia granted ethical approval. Principles of informed consent and confidentiality adhered to.<br>-Participants were not paid but transport costs were reimbursed<br>-Data were entered into NVIVO v.7, transcripts coded inductively thematically analysed.<br>-Two authors discussed ambiguous data and reconciled divergent interpretations.**	**Informational, psychosocial, sexual, and reproductive health (SRH)needs of adolescents living with HIV.**	**Themes:<br>1.Informational needs<br>Respondents expressed their need for information on family planning and prevention of STIs including HIV, because information from home were restrictive and messages from schools and clinics were laced with myths and stigma<br>2.Psychosocial and disclosure needs<br>This was needed because young people felt less connected and isolated due to non-disclosure from parents and stigma.<br>*"the fact my family kept this from me made me very sad; [for a time] I hated my mother'' (female, aged 19 years).*<br>Expressed need for support groups which focuses on social and vocational skills.<br>3.Sexual and reproductive health (SRH) needs of adolescents.<br>Culture and traditions affected need for privacy in clinics.<br>4.Health systems and services capacity:<br>Need for friendly health care workers, Availability of commodities because of work load, negative HCW attitudes and under-resourced clinics**

A

Study reference	Title	Year	Authors	Setting	Sample	Aims	Study Design	Phenomenon Explored	Findings
16 (eligible)	Young people's perception of sexual and reproductive health services in Kenya	2014	Godia, P.M., Olenja, J.M., Hofman, J.J. and Van Den Broek, N.	Involved 4 regions: the capital city of Nairobi and three districts (Laikipia, Meru Central and Kirinyaga).<br>-A total of 9 facilities were purposefully selected with the aim of including interviews with youth from facilities offering youth-only services as well as from facilities offering integrated SRH services.<br>-Facilities selected in Nairobi included five health facilities offering integrated services and one community-based youth centre.<br>-At the district level, the sample included 2 district hospitals which had facility-based youth centres (Laikipia and Meru) and 1 district hospital where SRH services for young people were integrated with regular health service provision.	-Young people (male or female, aged 10–24 years) seeking SRH services at a health facilities and community	To explore the experiences and perceptions of young people in Kenya aged 10–24 regarding their SRH needs and whether these are met by the available healthcare service	-Qualitative<br>-Used Focus group discussions (FGD) and semi-structured in-depth interviews in English and Kiswahili languages.<br>-Purposive sampling and snowballing were used for recruitment<br>-Ethical clearance and approval were obtained from the Liverpool School of Tropical Medicine Research Ethics Committee in the United Kingdom and the Kenyatta National Hospital Ethics and Research Committee in Kenya. Other ethical principles such as confidentiality, informed consent and anonymity ensured<br>-Data collection and analysis was sufficiently rigorous. Triangulation used<br>-Data transcribed verbatim, coded and thematically analysed.	Experiences and perception of sexual and reproductive health services	Themes:<br>1.SRH problems faced by young people: This included cultural, social, and economic environment in which they live in.<br>-Problems were same for boys and girls and included: early and unprotected sex, unwanted pregnancy, infection with STIs including HIV/AIDS, unsafe abortion, sexual violence, and female genital mutilation.<br>-Other concerns included inadequate information on reproductive health generally.<br>-problems related to physical body change during the period of adolescence and relationship problems. Social problems which young people considered important, included lack of parental guidance on sexuality and growing up, poverty and unemployment, drug, and substance abuse, media influence and peer pressure.<br>-Problems mentioned infrequently included prostitution, early marriage, and school drop-out<br>*"If we talk about services here [integrated health centres in Nairobi], mostly we will not talk about youths, we will mostly talk about mothers.....we can say the services here are smart.......on the side of mothers the services are perfect" (FGD boys, Nairobi)*<br><br>2.Addressing the SRH needs of young people: Young people generally wanted more accurate SRH information especially from parents on use of contraception including condoms and traditional methods (safe days) as ways of preventing unwanted pregnancy. With regards to STI and HIV/AIDS prevention, condom use, abstinence and not having many sexual partners<br>3.Perceptions of existing SRH services: Viewed services available at

A

Study reference	Title	Year	Authors	Setting	Sample	Aims	Study Design	Phenomenon Explored	Findings
									**the integrated health centres were serving the needs of women and children well.<br>-Girls described how "simple" things really mattered to them such as: HSP reception, facial expressions, simple greetings, being given the chance to express themselves and explain their problems. Most boys and girls were concerned about long queues that were present at some healthcare facilities. Boys reported being impatient and wanting "quick attention" without being lectured at or "tossed" from one hospital department to another<br>There was need for improvement in most public health facilities such as facility renovation, better HCW's attitude/approach and relationship with clients.<br>-Public health facilities were perceived to have more qualified staff compared to some private health facilities in urban slum areas.<br>-Had preferences for centres with recreational activities and provision of information, vocational training, and advice on career progression.<br>*"I think it is a good place for us to go because it is not like the other place [–general health facility -]…where most people go but this one [–youth centre-] is for the youths only and some of the staff working there are youths, like us, and they understand what we go through, so it is easier working with them", (IDI 14 year old girl, Meru"*<br>*"They provide everything, I can go there to play, to watch movies, I can be guided, I can be tested", (IDI 14 year old, Laikipia youth centre*<br>-Wanted opportunity to receive computer training, learn how to access the internet and write CVs.<br>4.Suggestions on how to improve SRH services<br>-Majority wished to see an increase in SRH services especially in rural**

A

Study reference	Title	Year	Authors	Setting	Sample	Aims	Study Design	Phenomenon Explored	Findings
									**areas including the use of mobile clinics. The consensus was that providing a wide range of SRH services in either integrated health facilities or youth centres was more likely to ensure anonymously, and that privacy could be maintained. Girls also mentioned having girl-talks, girls' days and "things to do with beauty" as a way of attracting girls.<br>-need to increase awareness of available SRH services among young people through outreach activities in the community, schools, and churches. Use of local radio stations, posters, magazines, sporting activities and entertainment.<br>-Having up to date educational materials at health facilities, libraries and other social places which young people frequent was also seen as helpful**

A

Study reference	Title	Year	Authors	Setting	Sample	Aims	Study Design	Phenomenon Explored	Findings
17<br><br>(eligible)	Understanding sexual and reproductive health needs of adolescents: evidence from a formative evaluation in Wakiso district, Uganda	2015	Atuyambe, L.M., Kibira, S.P., Bukenya, J., Muhumuza, C., Apolot, R.R. and Mulogo, E	-2 counties of Kyaddondo and Busiro, Wakiso district, central Uganda were randomly selected.<br>-3 sub-counties (names not mentioned) from each of the 2 counties were then selected ensuring inclusions of both peri urban and rural localities.<br>-According to the authors, most of the population is rural while the small urban and peri urban population is heavily influenced by city lifestyle with proximity to Kampala city. Wakiso has two counties and one municipality, 17 sub-counties and 131 parishes.	-A total of 156 adolescents participated aged 10-19years<br>- 4 FGDs in each of the 3 sub-counties were conducted with adolescent girls as well as boys (in and out of school) irrespective of whether they were seeking care or not.<br>-The selection was to ensure intra group homogeneity since the two groups of adolescents have been shown to exhibit different behaviours regarding health	To assess the sexual reproductive health needs of the adolescents and explore their attitudes towards current services available	-A qualitative study.<br>-20 FGDs stratified by gender (10 out-of-school, and 10 in-school).<br>-Purposive sampling used.<br>-Trained research assistants (moderator and note taker) used a pretested FGD guide translated into the local language to collect data.<br>-Ethical clearance was obtained from Makerere University School of Public Health Research and Ethics Committee and approval from the Uganda National Council of Science and Technology (UNCST).<br>-Informed consent obtained and assent obtained for participants < 18years<br>-All discussions were audio taped and were transcribed verbatim before thematically analysed using Atlas ti. Version 7.<br>-Data collection and analysis was sufficiently rigorous.<br>-No mention of specific method of rigor.	Understanding sexual and reproductive health needs of adolescents.	Themes:<br>1.Main adolescent health problems:<br>-The most important health problems expressed were HIV/STIs (all FGDs), unwanted pregnancies, sexual advances for the females from adult males and fellow male adolescents (expressed by female adolescents mostly), defilement and rape and the use of alcohol and other substances.<br>*"Most of the youths don't have what to do - they resort to taking alcohol, opium, cigarettes and marijuana. Even girls think that it is the today's style to put on thin trousers called 'skin tight'. And in so doing they meet these boys who have taken opium along the roadside and they force them into sex. In every town there are places like clubs and bars where youths take alcohol. (Males out of school)*<br>2.Adolescent SRH needs: Need for condoms to be put in accessible places at no cost, youth counsellors, teenage medical centres and post abortion care services was expressed.<br>*"…the need for condoms, you can go to the health facility and they give them to you but there are some people who do not know how to use them. He will just put it on anyhow. It requires that there is someone who gives them out but he/she first educated you on how to use them and he tells you that "I have given you that thing, use it like this. But there are those who just have them for showing off; they tell their friends that they have them" (Males out of school)*<br>*"The problem we have pertaining condoms e.g. life guard, some are expensive especially in the shops and the cheap ones are not available. So we young people end up having sex without condoms. (Males in school)*

A

Study reference	Title	Year	Authors	Setting	Sample	Aims	Study Design	Phenomenon Explored	Findings
									**Adolescents reported Another problem *I see as a young person is among the girls, they have aborted a lot. They don't get good counselling or advice on how they can protect and take care of the pregnancies. So they end up aborting" (Males in school*)** **Adolescents expressed that these needs were because of some of their peers who had used traditional dangerous methods and other unsafe means to get rid of unwanted pregnancies even with knowledge of consequences associated with these means including death. Also, some reported that some health workers gave them medicines to take home in case they wanted to abort or even help with illegal abortions.** ***''I was told that there are some girls who get some medicines from health workers. It is in form of tablets. When they swallow it, it gives them labour like pain, then they deliver and the pregnancy is terminated''. (Males out of school)*** **3.Health seeking behaviour and attitudes towards services: The authors found that adolescents when faced with RH problems take no action. It is only later when these problems persist that they visit a health facility.** ***"When we get problems, sometimes we tell our friends. Me I have a friend who has Candida, but she told us her friends and we inquired from an older person about it, who told us about some local medicine so that is what she is using. She had been to Kasangati health centre and they told her that the drugs are not there, they just wrote a prescription and they told her to buy the drugs from a clinic yet she had no money and no parents. So sometimes you can tell friend or neighbours, they may be of help. (Females in school)***

A

Study reference	Title	Year	Authors	Setting	Sample	Aims	Study Design	Phenomenon Explored	Findings
									**-Reasons for this poor health seeking behaviour reported included cost, privacy issues and the long queues due to very few health workers serving large populations. Some adolescents in male FGDs reported “nosy” health workers who they thought asked many questions making them uncomfortable. Also nonflexible opening and closing hours of facilities, lack of necessary drugs for STIs and other SRH problems, few health workers yet adolescents feared to open up to health workers of the opposite sex, and mistrusting of the health workers by the adolescents<br>-Some of the adolescents reported seeking help from traditional healers or used herbs.<br>-The overall quality of SRH services at the facilities was reportedly of poor quality to most of them as reported in fifteen of twenty FGDs.<br>4.Preferred services and modalities for their provision: Adolescents needed dedicated teenage health centre equipped with youth friendly health workers and stocked medicines. Adolescents also wanted provision of adolescent counselling services and health education. In health facilities outside of the teenage centre, adolescents want separate services to ensure privacy for them<br>-Regarding modalities of service provision, the adolescents preferred those services be available all the time (opening and closing hours), by younger health workers and of the same sex and in places that ensure privacy.<br>-Out-of- school male adolescent FGDs preferred outreach community services to be provided at no cost and preferably with health workers not from the same area.**<br>***“Another problem is that most of the health workers here are females***

A

Study reference	Title	Year	Authors	Setting	Sample	Aims	Study Design	Phenomenon Explored	Findings
									(they laugh). Such ladies are easy to their fellow ladies but to us boys it is not easy. (Males in school)<br>**-*In-school* adolescents preferred friendly health care workers and reduction in waiting time at the health facilities.**<br>***"Now what I am saying is that we as the youth we should get a special day say like the weekend and we have counselling and guidance, it will help us. (Males in school)***<br>***We [the adolescents] need health talks because there are some problems we face when we totally have no idea on how to go about them. So we should be health educated such that we know what to do. (Females out of school)***

A

Study reference	Title	Year	Authors	Setting	Sample	Aims	Study Design	Phenomenon Explored	Findings
**18**<br><br>**(eligible)**	**Adolescent human immunodeficiency virus self-management: Needs of adolescents in the Eastern Cape**	**2021**	**Adams, L. and Crowley, T**	**-2 primary healthcare clinics in the Nelson Mandela Bay area (NMBD) of the Eastern Cape.**<br>**-The clinics are situated in the Northern areas of the NMBD and fall under the sub-district C area. The clinics were selected based on the number of adolescents accessing ART and accessibility to the researcher.**	**-13 older adolescents between the age of 14 and 19 years participated in the study.**<br>**-Older adolescents (secondary school age) were chosen as opposed to younger adolescents because, based on their cognitive developmental stage. They are more capable of self-management skills such as self-monitoring, planning, goal setting and evaluation. It is also the age at which parents tend to transfer responsibility of care to the adolescent.**	**To explore the self-management needs of ALHIV in the Nelson Mandela Bay area of the Eastern Cape**	**-Used a qualitative exploratory-descriptive research design**<br>**-Purposive sampling was used to sample participants across different ages, languages, and genders.**<br>**-A semi-structured interview guide was used and was pilot tested.**<br>**-Ethical approval was obtained from the Health Research Ethics at the University of Stellenbosch, and permission was obtained from the Eastern Cape Department of Health.**<br>**- Confidentiality and anonymity ensured. Informed consent sought**<br>**-13 individual interviews were conducted, and the sample size was determined by the emerging themes in the interviews and data saturation.**<br>**-Interviews were recorded on an audio-recorder. Interviews were conducted in Afrikaans or English.**<br>**-Interviews were transcribed verbatim and checked by the first author.**<br>**-Data analysis was done using the six steps described by Creswell.**<br>**-Prolonged engagement, reflexivity, recording of information and member checking were used to ensure credibility of data.**	**Self-management needs of ALHIV**	**Themes:**<br>**1.Knowledge of human immunodeficiency virus and sexual reproductive health:**<br>**-lacked knowledge about HIV and sexual reproductive health. Participants were uncomfortable to talk to caregivers or guardians about HIV, sex or matters regarding life. They, therefore, accessed information about HIV by using the internet on their mobile phones or at home. Positive feelings, such as hope, emanated from their acceptance of the illness.**<br>**2.Self-regulation skills: Included decisions about disclosure, managing stigma, taking treatment, managing emotions,**<br>***"It will hurt if other people know, they will joke about it.' (Participant 10, 14-year-old male)***<br>***'I felt so hurt because I didn't know.' (Participant 7, 14-year-old female) 'I felt very hateful at first.' (Participant 6, 19-year-old female)***<br>**communicating, and setting goals as key self-regulation skills.**<br>**3.Self-management resources: The resources that the participants used for social facilitation included health facilities, relationship with health care workers, Support from family and friends.**

A

Study reference	Title	Year	Authors	Setting	Sample	Aims	Study Design	Phenomenon Explored	Findings
19<br><br>(eligible)	Preferences for accessing sexual and reproductive health services among adolescents and young adults living with HIV/ AIDs in Western Kenya: A qualitative study	2022	Adhiambo, H. F, Ngayo, M. and Kwena, Z.	-Lumumba Sub-County and Kisumu County hospitals in Kenya.<br>-The two hospitals are public hospitals with HIV care programs supported by Family AIDS Care and Education Services (FACES) and have a combined HIV patient population of 14,661, with 9.5% being adolescents and young adults (AYAs).	-AYAs aged 14–24 years, healthcare workers, and caregivers/parents.<br><br>-Adolescents and young adults were eligible if they were aged 14–24, living with HIV and on care at the two hospitals.<br>-30 AYAs participated in the study.<br>-Most participants were female (57%), aged between 15 and 19 (73%). One-third had completed secondary education, while others had some secondary or college-level education. Regarding occupation, the majority (63%) were still in school, and one-fifth were unemployed. Only 16% reported employment, with 3% being self-employed. More than half of our participants (57%) had one/both parents deceased, with 27% with both parents deceased. Most participants (97%) were single, with 80% sexually active and only 7 had a pregnancy history	To explore adolescents', healthcare workers, and caregivers' preferences for access to sexual and reproductive health services for adolescents and young adults living with HIV	-Used a qualitative design. No mention of the specific qualitative design<br>-Purposive sampling was used to sample participants.<br>-30 In-depth interviews and 8 focus group discussions were conducted.<br>-Ethical approval was obtained from the Kenya Medical Research Institute's Scientific and Ethics Review Unit (KEMRI/SERU/CMR/00064 /3528)<br>- Informed consent/assent sought. Privacy ensured. Confidentiality and anonymity ensured since authors stated that they adhered to Helsinki's declaration.<br>-Data was collected by Research Assistants trained in qualitative methods with over three years of experience working with AYA in HIV clinics and SRH clinics. e Research Assistants were 5–10 years older than the adolescents.<br>-Interviews were conducted in participant's preferred language (English, Swahili, or Dholuo). Both the IDI and FGD guides were available in English, Kiswahili, and the local Dholuo languages.<br>-The study was mainly conducted during school breaks and some during their clinic appointment dates.	Self-management needs of ALHIV	Themes:<br>1.Preferences of venue for receiving SRH services:<br>-The AYAs had differing perspectives on the ideal location for receiving SRH services. The venue to access the services varied from schools, health families, workplaces, churches, homes, youth-friendly centres, community halls, and hotels. Some of the critical considerations in the choice of venue to access SRH services were proximity, cost, waiting time at the venue, and concerns about privacy and confidentiality.<br>2.Preferences of qualities of SRH counsellors: AYAs preferred providers who were their agemates because they said that it was easier for a young person to open up to such a peer provider. They argued that the sameage peer provider would understand what they are going through and speak the same language. AYAs also pointed out that older people tend to misunderstand young people or ignore them altogether, thus creating an element of fear. However, others admired the seasoned knowledge, experience, and parental love that some older providers might exhibit towards young people.

A

Study reference	Title	Year	Authors	Setting	Sample	Aims	Study Design	Phenomenon Explored	Findings
							-Theoretical saturation determined the interviews.<br>-Audio recorded interviews were downloaded onto a password-protected computer, backed up on external drives, and kept off-site. The IDIs lasted approximately 40 while the FGDs went up to 90 minutes.<br>-Data transcribed verbatim and analysed using Nvivo.<br>-Constant comparative method was used to discover dominant themes that helped authors the perspectives of adolescents', healthcare workers', and caregivers' perspectives on AYAs living with HIV.<br>-Triangulated data sources.		

A

Study reference	Title	Year	Authors	Setting	Sample	Aims	Study Design	Phenomenon Explored	Findings
**20**<br><br>**(eligible)**	**Pregnancy and STI/HIV prevention intervention preferences of South African adolescent girls: findings from a cultural consensus modelling qualitative study**	**2023**	**T. Dylanne Twitty, Anthony E. Hitch, Lochner Marais, Jessica M. Sales, Carla Sharp, Jan Cloete, Molefi Lenka, Kholisa Rani, Nicole K. Gause & Jennifer L. Brown**	**Mangaung Metropolitan Municipality in the Free State Province of South Africa. Unclear why the setting was considered for the study.**	**-Sesotho-speaking adolescent girls and young women aged between 14 and 17.<br>-All participants reported being currently enrolled in government funded public schools, with the majority reporting no current employment.**	**To explore pregnancy and STI/HIV prevention intervention preferences of South African adolescent girls in Mangaung Metropolitan Municipality, South Africa.**	**-Used a qualitative design. No mention of the specific qualitative design.<br>-Study protocols were approved by the University of the Free State Institutional Review Board.<br>-Parental consent and assent obtained. Confidentiality and anonymity ensured<br>-Purposive sampling was used to sample participants.<br>-Semi-structured interviews were conducted in Sesotho language.<br>-interviews were digitally recorded, transcribed verbatim, and translated into English.<br>-Used conventional content analysis approach using NVivo**	**Pregnancy and STI/HIV prevention intervention preferences of South African adolescent girls:**	**Themes:<br>1.Intervention content:<br>-Participants indicated a desire to learn more about STI/HIV *''I think the most important topic will be pregnancy and STI and HIV prevention''*<br>*'' People are no longer given deep information about these things [sexual health], they are only given average information''*<br>prevention strategies such as condom use, in-depth sexual health information.<br>2.Intervention delivery format: Preferred intervention delivery formats were those that (a) were easily accessible (e.g. online, - *"They would prefer receiving it online because that is where they will receive all the information they need" (Ethel)* text/SMS), (b) provided high-quality information, and (c) were delivered by knowledgeable, skilled facilitators. Emphasis were on knowledgeable, skilled facilitators to deliver sexual health interventions who would ensure that confidentiality was protected and create a safe, non-judgemental environment for sexual health discussions. Preferred facilitator characteristics were being trustworthy, well-informed, and providing non-biased sexual health recommendations.<br><br>3.Intervention setting: The most common considerations for choice of setting were a) familiarity and accessibility, b) concerns regarding negative judgement, and c) access to trusted information. Participants had interest in locations that already provide sexual health information and resources such as existing clinics, youth centres, schools and homes, with the most preferred being school and clinics.**

Data extractors – Victoria Uka (VU) and Debbie Smith (DS)

Period of Data extraction- November 2022 – August 2023
