# Supplementary figures and images for: The sexual and reproductive health needs and preferences of youths in sub-Saharan Africa: A meta-synthesis
Source: PLoS One. 2024 Dec 31;19(12):e0300829. doi: 10.1371/journal.pone.0300829 (PMC11687907; doi:10.1371/journal.pone.0300829)

S5- PICO framework for review question and search terms

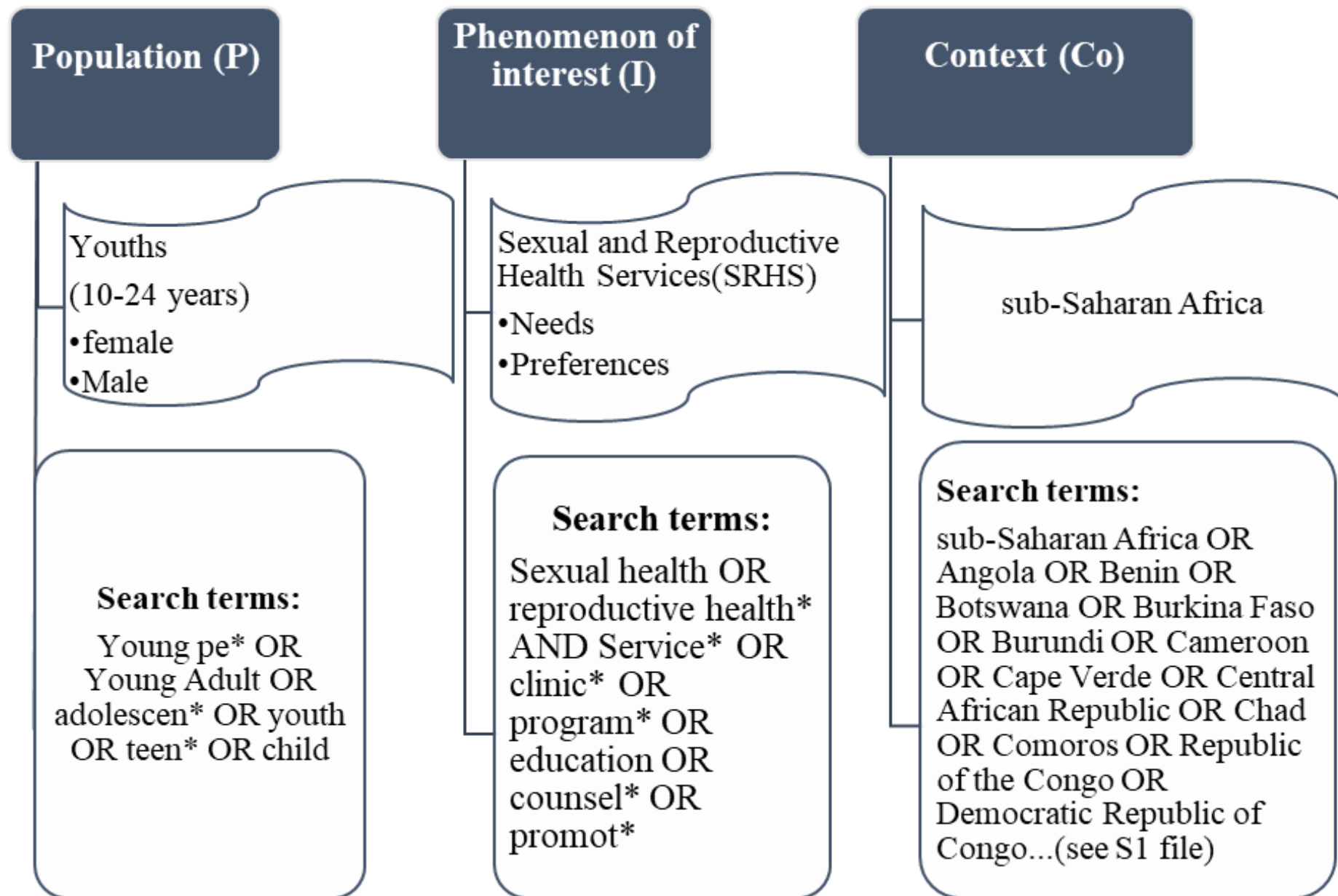

Supplement: S1 Fig — (PDF) [file pone.0300829.s007.pdf]

**S6- PRISMA flow diagram showing stages of study selection**

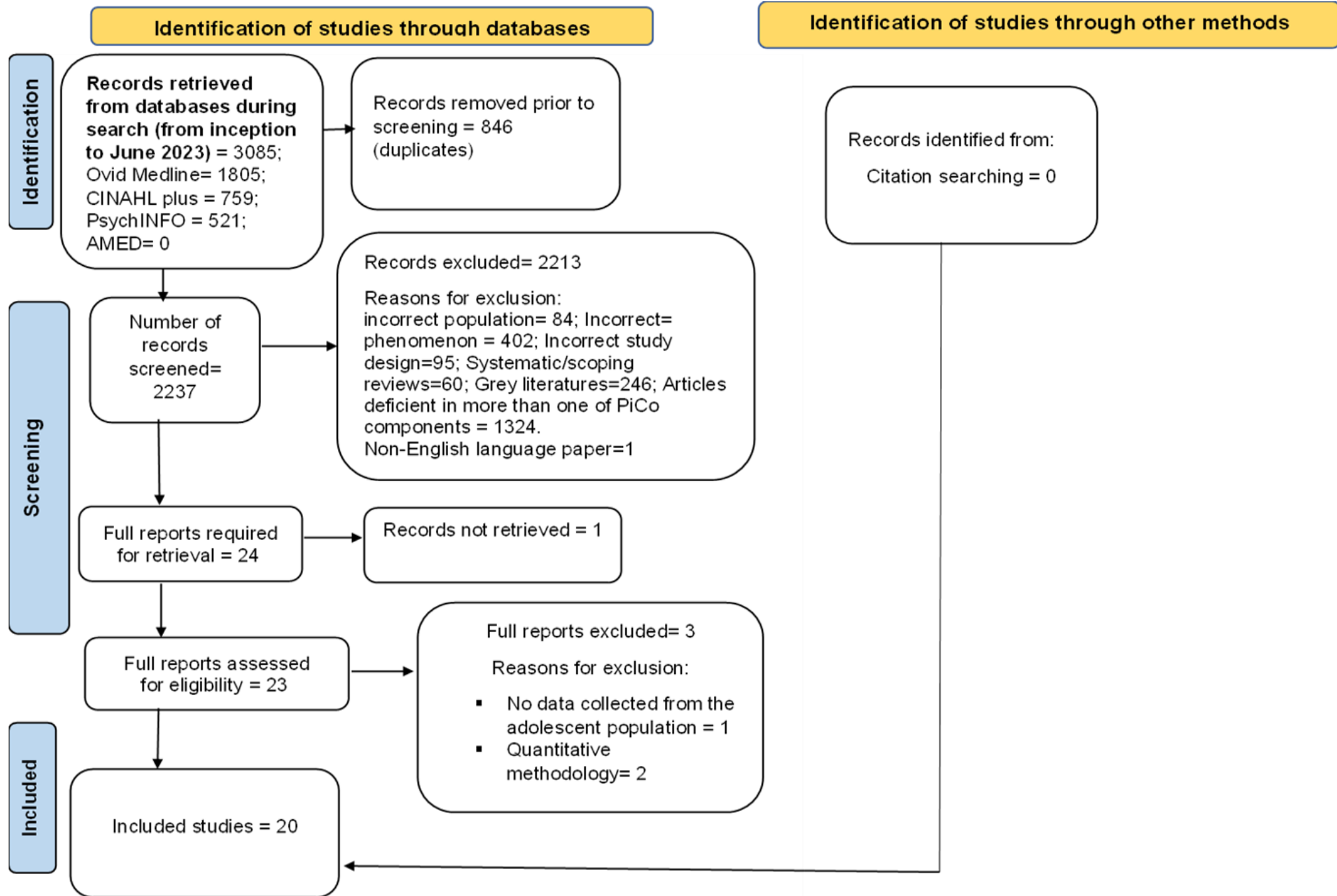

Supplement: S2 Fig — (PDF) [file pone.0300829.s008.pdf]
