# Supplementary material for: The sexual and reproductive health needs and preferences of youths in sub-Saharan Africa: A meta-synthesis
Source: PLoS One. 2024 Dec 31;19(12):e0300829. doi: 10.1371/journal.pone.0300829 (PMC11687907; doi:10.1371/journal.pone.0300829)
Supplement: Attachment — Submitted filename: mynotes_PONE_20240524.docx [file pone.0300829.s009.docx]

| lines | text | Reviewer’s comment |  |
| --- | --- | --- | --- |
| 61-67 | “…” | This is the paragraph for the reader to understand the magnitude of the problem you are dealing with. The proportion of what happens in SSA is missing and not easily understandable. For instance, it is not understandable whether the figures on 374 million STIs cases occur worldwide in L&MIC or are referred to SSA L&MIC; same for 21 million pregnancies. If these figures are worldwide, can you provide a proportion of those occurring in the study area? |  |
| 124 | “Grey literature was excluded as they can be difficult to comprehensively identify and  124 access, introducing selection bias if relevant reports are missed [35].” | [35] aims to valorise grey literature, yet it is reported to justify grey literature exclusion. [35] underlines risk of publication biases due to grey literature exclusion. Authors refer a selection biases risk reported by [35] which is quite generic. Authors should be more specific on why they excluded grey literature and why it was not possible to compensate biases introduced by grey literature with the commercially and open publications. I would encourage Authors to evaluate the content of GL they excluded for other insights other than those from the review of 20 papers |  |
| 157 | “For each question,  157 the responses "yes," "can't tell," and "no" were assigned scores of 2, 1, and 0, respectively. | The scoring does not add any information to the qualitative categorisation, then as such, it is redundant |  |
| 213-214 | Nevertheless, no studies were excluded solely based on quality, as the authors were primarily interested in extracting relevant concepts from all included studies that directly mapped to the systematic review question. | CASP checklist checks the scientific quality of the study among other features, if the CASP checklist result was ignored why did you exclude grey literature because of “selection biases”? You may have found “relevant concepts from all included studies that directly mapped to the systematic review question” in GL as well. |  |
| 234 | Fig 3: Showing main themes and sub-themes | A pie graph is generally referred to something describing an entire. As such this figures seems indicating that one fourth of the entire SRHS is captured by each of 4 themes, which is not the case as the 4 themes are the key (major?) themes and implies there are other minor themes . Authors should visualise the themes and subthemes differently, a table will be fine. |  |
| 372-373 | “In line with this, our review  found that guided education enables youths to develop healthy sexuality perspectives and protects against risky sexual and reproductive behaviours.” | Reviewed papers are not RCT that tested interventions (i.e. guided education) to estimate whether it “enable youth…”, then this review cannot have “found”. You should rearrange the sentence |  |
| 451 | Strength and limitations | No limitation are mentioned (referring to broader than SSA review is not a limitation given that the study area is well declared in title and methods). Not citing grey literature data exclusion is a limitation for a topic that may have addressed the government/institutions attention to a problem that is related to big management issues such as drug abuse, violence, HIV,… |  |
