# Supplementary material for: The sexual and reproductive health needs and preferences of youths in sub-Saharan Africa: A meta-synthesis
Source: PLoS One. 2024 Dec 31;19(12):e0300829. doi: 10.1371/journal.pone.0300829 (PMC11687907; doi:10.1371/journal.pone.0300829)
Supplement: Attachment — Submitted filename: Response to Reviewers.docx [file pone.0300829.s010.docx]

Mrs. Victoria Kalu Uka

The University of Manchester

Division of Nursing, Midwifery and Social Work

Manchester

M13 9PL

[victoria.uka@postgrad.manchester.ac.uk](mailto:victoria.uka@postgrad.manchester.ac.uk)

10^th^ June 2024

The Editorial Office

PLOS One

Dear Editors,

We greatly appreciate the insightful comments provided by the reviewers on our manuscript titled “The Sexual and Reproductive Health needs and preferences of youths in sub-Saharan Africa: A meta-synthesis”. We have carefully considered each point raised and have revised the manuscript accordingly. Please find our detailed responses below:

Response to Reviewer's Comments:

Comment 1 (Lines 61-67): This comment sought clarification on the magnitude of the problem in sub-Saharan Africa (SSA) concerning sexually transmitted infections (STIs) and pregnancies among youth. We have addressed this by:

1. Clarifying that the 374 million new cases of STIs occur globally, with a significant proportion in low-and middle-income countries (LMICs), and emphasising the considerable share borne by youth in SSA.
2. Highlighting that approximately 50% of the 21 million pregnancies among 15-19-year-olds in LMICs occur in SSA, thus providing a clearer understanding of the problem's magnitude in the study area.
3. Enhancing the overall narrative to better reflect the specific challenges faced in SSA, addressing gender inequality and its impact on sexual and reproductive health.

Comment 2 (Line 124): The reviewer questioned the exclusion of grey literature and suggested evaluating its content for additional insights. We have revised our manuscript to provide a more detailed justification for the exclusion of grey literature, acknowledging the potential for publication bias. However, we chose to exclude grey literature due to difficulties in comprehensively identifying and accessing these sources, the heterogeneous nature of grey literature, and the potential lack of rigorous peer review, which could affect the integrity of our meta-synthesis. We prioritised published, peer-reviewed studies to ensure the quality and reliability of our sources. Nonetheless, we recognise that excluding grey literature might introduce some bias, and in future research, we may consider evaluating the content of excluded grey literature for additional insights.

Comment 3 (Lines 157): The reviewer questioned the necessity of the scoring system used in the quality appraisal section. We have clarified that the scoring system aimed to enhance transparency and consistency in our quality assessments. Assigning scores facilitated inter-study comparisons and supported our overall quality judgments. Importantly, no studies were excluded based on their scores; all papers were included for their valuable contributions to the meta-ethnographic synthesis. We have revised the quality appraisal section to reflect this explanation.

Comment 4 (Lines 213-214): The reviewer questioned our rationale for excluding grey literature despite not excluding studies solely based on the CASP checklist quality scores. Our decision to exclude grey literature was primarily driven by several considerations:

1. Comprehensive identification and access: Grey literature can be challenging to comprehensively identify and access. Unlike peer-reviewed studies, grey literature is often dispersed across various platforms and lacks standardised indexing, making it difficult to ensure a thorough and systematic search.
2. Quality and rigorous peer review: One of our main aims was to ensure the scientific quality and reliability of our sources. Peer-reviewed studies undergo rigorous review processes that help ensure the validity and replicability of findings. In contrast, grey literature often does not undergo such stringent peer review, posing challenges in assessing its scientific quality.
3. Heterogeneous nature: Grey literature is highly heterogeneous, varying widely in terms of format, quality, and scope. This heterogeneity complicates the assessment and synthesis of findings, which could affect the overall integrity of our meta-synthesis.

Regarding the CASP appraisal, while we did not exclude studies solely based on their CASP checklist scores, we did use these scores to inform our understanding of each study's methodological rigor. The inclusion of studies, regardless of their quality scores, was aimed at capturing a wide range of relevant concepts. However, we prioritised published, peer-reviewed studies to maintain a high standard of quality in our review. We acknowledge that excluding grey literature might introduce some bias. In future research, we may consider evaluating the content of excluded grey literature to pick up additional insights that could complement our findings. This approach was necessary to maintain the integrity and trustworthiness of our meta-synthesis. We have documented this rationale in an earlier comment (comment 2, line 124) and have ensured a comprehensive collection of relevant studies by thoroughly searching commercial and open-access databases focusing on the sexual and reproductive health needs and preferences of youths in sub-Saharan Africa.

Comment 5 (Line 234): Regarding the visualisation of the main themes and sub-themes, we have replaced the pie chart with a more appropriate table format to avoid any misinterpretation and ensure clarity in representing the findings.

Comment 6 (Lines 372-373): The reviewer suggested rephrasing the statement about guided education enabling youth to develop healthy sexuality perspectives. We have revised the sentence to: “Our review suggests that guided education may be associated with the development of healthy sexuality perspectives and potentially protective against risky sexual and reproductive behaviours among youths.”

Comment 7 (Line 451): The reviewer mentioned that no limitations were stated in the manuscript, particularly regarding the exclusion of grey literature data for a topic that may have addressed government and institutional responses to significant public health challenges such as drug abuse, violence, and HIV. We have revised the limitations section to acknowledge the limitation of not citing grey literature data, which could provide valuable insights into government and institutional responses to significant public health challenges such as drug abuse, violence, and HIV.

We hope that our responses and revisions have addressed the concerns raised by the reviewers and further strengthened our manuscript. We remain open to any additional feedback or suggestions for improvement.

Thank you for your consideration.

Sincerely,

Victoria Uka

PhD candidate- Division of Nursing, Midwifery and Social Work

School of Health Sciences, Faculty of Biology, Medicine and Health

University of Manchester, Manchester
