# Supplementary material for: The sexual and reproductive health needs and preferences of youths in sub-Saharan Africa: A meta-synthesis
Source: PLoS One. 2024 Dec 31;19(12):e0300829. doi: 10.1371/journal.pone.0300829 (PMC11687907; doi:10.1371/journal.pone.0300829)
Supplement: Attachment — Submitted filename: Response to Reviewers.docx [file pone.0300829.s011.docx]

Mrs. Victoria Kalu Uka

The University of Manchester

Division of Nursing, Midwifery and Social Work

Manchester

M13 9PL

[victoria.uka@postgrad.manchester.ac.uk](mailto:victoria.uka@postgrad.manchester.ac.uk)

20^th^ August 2024

The Editorial Office

PLOS One

Dear Editor and reviewer #3,

We thank you for your continued consideration of our manuscript ‘’The Sexual and Reproductive Health needs and preferences of youths in sub-Saharan Africa: A meta-synthesis’’ (PONE-D-24-08352R1). We appreciate the additional feedback provided in this second round of review and have carefully addressed each point raised by Reviewer #3. Below, please find our detailed responses to each comment.

Response to Academic Editor:

We thank the editor for acknowledging the improvements made in our previous revision and for guiding us to address the remaining concerns raised by Reviewer #3. We have carefully followed these suggestions to further enhance our manuscript.

Response to Reviewer #3:

Abstract:

1. Comment: Line 31: First use of CASP should be spelled out

Response: We thank the reviewer for this observation. We have spelled out CASP as ‘’Critical Appraisal Skills Programme (CASP)’’ on its first use in the abstract.

Introduction:

2. Comment: Line 56: consider modifying language – perhaps change ‘’including’’ to ‘’by’’ or ‘’due to’’

Response: We appreciate this suggestion and have changed ‘’including’’ to ‘’due to’’ to improve clarity.

3. Comment: Line 56-57: Suggest modifying or omitting sentence starting ‘’As a result’’

Response: We have modified this sentence to provide more specific information and improve the flow. The new sentence reads: ‘’These barriers contribute to significant disparities in youth SRHS access and utilisation across SSA countries.’’ This revision maintains the concept of inconsistency while adding more depth to our discussion of the challenges in SRHS access.

4. Comment: Line 59: not necessary to define the acronym SRHS again.

Response: We agree and have removed the redefinition of SRHS at this point in the manuscript.

5. Comment: Line 74: Purpose of citation [22] is unclear.

Response: We have reviewed the context of citation [22] and have modified the sentence to clarify its purpose. The revised sentence now reads: ‘’Healthy development is hindered [22] and potentially leads to substance misuse or mental health issues [23]’’. Citation [22] (Hegde, Chandran and Pattnaik, 2022) discusses adolescent development and sexuality from a developmental perspective. It emphasises that adolescence is a critical period for healthy development, including sexual development. The article supports our point that when sexual and reproductive health needs are not met, it can hinder overall healthy development in adolescents. So, to improve clarity, we have ensured that the connection between unmet sexual and reproductive health needs and hindered development is more explicitly stated in the surrounding text. This helps to better contextualise the purpose of citation [22] within our argument. This modification provides a clearer link between the citation and our argument about the importance of addressing adolescents' sexual and reproductive health needs for their overall development.

Methods:

6. Comment: Line 117: Consider replacing ‘’homeless’’ with ‘’houseless’’

Response: We appreciate your suggestion to replace 'homeless' with 'houseless'. However, we have decided to retain the term 'homeless' in our exclusion criteria for several reasons:

- It accurately reflects the terminology used in our original systematic review methodology.
- Changing the term retrospectively could potentially alter the perceived scope of our study.
- To maintain consistency with the broader literature in this field, which predominantly uses the term 'homeless' when discussing excluded populations in similar contexts.

While we acknowledge the evolving nature of terminology in this area, we believe that maintaining our original wording is crucial for the accuracy and reproducibility of our review. Thank you for bringing this important point to our attention.

7. Comment: Line 129-136: Please resolve redundancy involving ‘’grey literature’’- important to be concise.

Response: We had previously expanded this section based on feedback from another reviewer who questioned our exclusion of grey literature. However, we appreciate your emphasis on conciseness, and we have revised this section to provide a clear but more succinct explanation of our approach. We believe this revision maintains the necessary justification while improving the overall readability of our methods section.

8. Comment: Line 142: Suggest modifying as other sources of information ‘’may lack’’ or may not have a transparent peer review process.

Response: We have modified this sentence to read ‘’may lack a transparent peer review process’’ to more accurately reflect the variability in review processes.

9. Comment: Line 152-153: What was the Kappa level used to confirm agreement/reliability?

Response: We have clarified that while we described the process of using Cohen's kappa in the methods section, the specific Kappa values are reported in our results section. We have added a brief mention in the methods that specific Kappa values will be reported in the results.

10. Comment: Line 186: Best to be consistent with SRHS or SRH, if possible.

Response: We have reviewed the manuscript and standardised our use of ‘SRHS’ (Sexual and Reproductive Health Services) when referring to services, retaining ‘SRH’ only when discussing the broader concept of Sexual and Reproductive Health. This distinction allows us to be precise in our language while maintaining consistency. However, we have reviewed the manuscript to ensure consistent use of SRHS where applicable.

11. Comment: Line 209: independent researchers or by researchers, independently?

Response: We have clarified the phrasing about the inter-rater reliability check. The statement now reads: ‘’Prior to finalising the selection process, 10% check of titles and abstracts was conducted on the retrieved articles by two researchers independently (VU and LM) to compute an inter-rater reliability.’’

To address the redundancy you noted (Comment 13: Line 230-232), we have moved this statement to the methods section, replacing a similar but less comprehensive statement that was previously there. This consolidation helps to streamline our methodology description while ensuring all necessary information is retained. These revisions aim to improve clarity and conciseness in our reporting of the review process. We believe these changes address your concerns about both the phrasing and the structure of our methodology description.

12. Comment: Line 218: It is very odd that a peer-reviewed article was not accessible – Is it possible to elaborate further? It also seems inconsistent with the methods where inclusion was considered by title and abstract and full text.

Response: We have elaborated on our attempts to access this specific article and the barriers we encountered. Response: The article in question is: ‘’Reproductive health needs of young persons in markets and motor parks in Southwest Nigeria’’ (<https://europepmc.org/article/med/14510129>). While we were able to access and screen the abstract of this article during our initial review process, we encountered significant barriers in obtaining the full text:

- The article passed our initial screening based on its title and abstract, which aligned with our inclusion criteria.
- However, when we attempted to access the full text, we found it was not available through open access platforms or our institutional subscriptions.
- The article lacked any contact information for the authors, including email addresses, which prevented us from reaching out directly to request the full text.
- We explored all available avenues to access the full text through our institutional resources, but were unsuccessful in obtaining it.

Given our inability to access the full text despite these efforts, we had to exclude this article. We have rephrased the relevant section concisely to reflect this explanation. We believe this explanation addresses the apparent inconsistency you noted and provides a clearer picture of our comprehensive screening and selection process while maintaining transparency in our methodology.

13. Comment: Line 230-232: this is redundant - specified in methods.

Response: We have removed this redundant statement from the results section as it is already covered in the methods.

Discussion:

14. Comment: Overall, this section would benefit from additional specific recommendations and findings, where possible.

Response: We have addressed this by making the following changes:

- We have restructured the discussion to highlight key findings more explicitly where possible. For the first three subheadings of the discussion (Youth empowerment through targeted information, aligning SRH services to diverse youth needs, and addressing social needs for holistic SRHS), we now begin each section with a clear statement of a main finding. For example: ‘’A key finding of this review is that youths strongly desire age-appropriate sexual and reproductive health information, but often lack access to reliable sources.’’ ‘’Our findings reveal a significant mismatch between available SRH services and the diverse needs of youth in SSA.’’ ‘’Our review highlights the interconnectedness of social factors and SRH outcomes among youth in SSA’’
- We have included more specific examples from the included studies to illustrate our points. For instance, we now mention that ‘’in one study [75], youths reported seeking information from older siblings or friends, often receiving inaccurate or incomplete information,’’ and ‘’in one study [67], youths reported using makeshift condoms from bread bags due to lack of access to proper contraceptives.’’
- We have added a new ‘’Implications and recommendations for practice’’ section, which provides six specific, actionable recommendations based directly on our review findings. We believe these changes provide the additional specific findings and recommendations you suggested, enhancing the practical implications of our review.

Conclusion:

15. Comment: Line 489: First sentence is not really a conclusion

Response: We have revised the opening to more clearly state the main finding of our review. We believe this revision provides a stronger, more conclusive opening that directly states the primary outcome of our study.

16. Comment: Line 500-502: There is quite a lot of literature in this space – are you able to be more specific about the gap and/or the indicated "need" for additional research?

Response: We have revised the conclusion to provide more specific research directions based on the gaps identified in our review. We now specify three key areas for future research: (1) Developing and evaluating youth-centred SRHS delivery models, (2) Assessing the effectiveness of integrated services, and (3) Conducting implementation studies on scaling up promising interventions.

We believe these revisions have significantly improved our manuscript and hope that it now meets the standards for publication in PLOS ONE. We look forward to your response and are happy to address any further questions or concerns.

Sincerely,

Victoria Kalu Uka

On behalf of all co-authors
